# Psychometric properties of the Burnout Assessment Tool across four countries

**DOI:** 10.1186/s12889-023-15604-z

**Published:** 2023-05-04

**Authors:** Kleinjan Redelinghuys, Brandon Morgan

**Affiliations:** Department of Industrial Psychology and People Management, University of Johannesburg, Johannesburg, South Africa

**Keywords:** Bifactor modelling, Burnout, Burnout Assessment Tool, Differential item functioning, Maslach Burnout Inventory, Psychometric properties, Rasch model

## Abstract

**Background:**

The Burnout Assessment Tool (BAT) is a new burnout measure developed to replace the Maslach Burnout Inventory (MBI). Studies have supported the psychometric properties and cross-cultural measurement invariance of the BAT. However, some unresolved questions remain. These questions are the appropriate level of score interpretation, convergent validity with the MBI, and measurement invariance using sample groups from countries outside of Europe.

**Methods:**

We used a cross-sectional survey approach to obtain 794 participants from Australia (*n* = 200), the Netherlands (*n* = 199), South Africa (*n* = 197), and the United States (*n* = 198). In brief, we used bifactor modelling to investigate the appropriate score interpretation and convergent validity with the MBI. Hereafter, we used the Rasch model and ordinal logistic regression to investigate differential item functioning.

**Results:**

The bifactor model showed a large general factor and four small group factors, which suggests calculating and interpreting a general burnout score. This model further shows that the BAT and MBI measure the same burnout construct but that the BAT is a more comprehensive burnout measure. Most items fit the Rasch model, and few showed non-negligible differential item functioning.

**Conclusions:**

Our results support the psychometric properties and cross-cultural measurement invariance of the BAT in Australia, the Netherlands, South Africa, and the United States. Furthermore, we provide some clarity on the three previously mentioned unresolved questions.

**Supplementary Information:**

The online version contains supplementary material available at 10.1186/s12889-023-15604-z.

## Introduction

The Burnout Assessment Tool (BAT) is a new burnout measure developed by Schaufeli et al. [61] to replace the Maslach Burnout Inventory (MBI). A 23-item version—the focus of our study—and a shorter 12-item version exist. The BAT measures four burnout dimensions: (a) Exhaustion, (b) Mental Distance, (c) Cognitive Impairment, and (d) Emotional Impairment (defined later in the article). Research supports the psychometric properties and cross-cultural validity of the BAT—at least with sample groups from Europe (e.g., [17])—meaning that practitioners and researchers can use it to obtain burnout scores. However, some unresolved questions with implications for the BAT exist. These questions are (a) the appropriate level of score interpretation, (b) convergent validity with the MBI, and (c) measurement invariance for countries outside of Europe. We expand on these questions and their importance below.

### The appropriate level of score interpretation

The BAT has two scoring options: a general (summated total) burnout score or four dimension scores [61]. Support for these options comes from the fit of second-order factor models (e.g., [72]), which operationalises the general burnout factor as the shared variance between the first-order factors (see [43]). However, this model might not be the best approach to deciding which scores to interpret because it can potentially lead to erroneous conclusions about the viability of group factors [11]. Following Reise et al. [53], we believe that a bifactor model and its associated unidimensional indices [55, 56] can better determine appropriate score interpretation.

We know of two studies that have applied a bifactor model to the BAT items, both of which argued in favour of a general burnout score [16, 58]. However, they did not report on all the unidimensional indices, making it difficult to determine which scores to calculate and interpret. This uncertainty is a problem because using the wrong scores can lead to a loss of statistical information and interpretational ambiguity [22, 23, 73]. For example, a summated Exhaustion score might reflect a combination with other three scales instead of just Exhaustion. It is important to also examine dimensionality from the perspective of predictive and incremental validity [22]. To our knowledge, no studies have investigated these validities for the BAT general burnout and group scores. Therefore, we also use Ferrando and Lorenzo-Seva’s [22] external validity approach to clarify which scores to calculate and interpret.

### The convergent validity of the BAT with the MBI

Convergent and discriminant validity are important sources of information on how one measure differs from others [1]. Regarding the BAT, convergence with the MBI will support its validity as a burnout measure. Discriminant validity will indicate that the BAT measures parts of burnout that the MBI does not capture. Researchers usually use the multitrait-multimethod (MTMM) approach to investigate convergent and discriminant validity [57]. Studies have supported these two validities for the BAT and MBI (e.g., [4, 48, 58]). The MTMM approach is useful but has limitations. One major limitation is convergence problems when estimated via factor analysis [21]. Instead of using the MTMM approach, we employ a bifactor model to investigate how much the items of the two measures converge (e.g., [16]). From this perspective, convergent and discriminant validity means that the BAT and MBI items measure the same or different constructs at the general factor and group levels (see [9, 39, 63]).

### Measurement invariance of the BAT

Measurement invariance, or fairness, means that the measurement properties of a measure are the same across groups, a requirement for cross-group score comparisons [10]. Studies generally support the cross-cultural measurement invariance of the BAT (e.g., [17, 65]). As of writing this article, de Beer et al. [17] have conducted the most comprehensive measurement invariance study. They found invariance with sample groups from the Netherlands, Belgium, Germany, Austria, Finland, Ireland, and Japan. Six of these countries are members of the European Union. Speculatively, these countries might share similar economic, political, and labour contexts that affect the meaning people attach to burnout and how they respond to the BAT items (see [59]). We use participants from Australia, the Netherlands, South Africa, and the United States to understand further the measurement invariance of the BAT items in three countries outside of Europe and to build on the results from de Beer et al. [17].

Most BAT measurement invariance studies have relied on second-order factor models (e.g., [17]). A second-order model provides valuable information on the invariance of relevant item parameters at different measurement levels. However, its focus is on a measurement model as a whole rather than the individual items. Instead, we use differential item functioning from a Rasch and ordinal logistic regression perspective because it provides greater clarity and a more nuanced understanding of how each item functions across groups [28]. A benefit of using the Rasch model is that it also provides information on the measurement properties of the BAT items that cannot be obtained by factor analysis (e.g., [31, 49]).

### Research questions

Against this background, we set out to determine (a) the appropriate score interpretation and (b) the convergent validity of the BAT with the MBI using bifactor modelling, and (c) investigate differential item functioning of the BAT item scores across four countries using the Rasch measurement model and ordinal logistic regression.

### The development of the BAT

The BAT is a 23-item measure of burnout developed by Schaufeli et al. [61] to replace the MBI, the most used and well-known burnout measure. They identified three major problems with the MBI. First, it has “conceptual, technical and practical imperfections” (p. 7); second, it “was developed almost forty years ago” (p. 7); and third, it is a research rather than an assessment measure of burnout. Schaufeli et al. [61] set out to reconceptualise burnout and create a measure of this reconceptualisation. They interviewed medical professionals and searched through existing burnout measures to achieve these objectives. Schaufeli et al. [61, 62] identified four core burnout symptoms, Exhaustion, Mental Distance, Emotional Impairment, and Cognitive Impairment, and three secondary dimensions, Psychological Distress, Psychosomatic Complaints, and Depressed Mood. We focus on the four core symptoms in our study.

Exhaustion is the loss of physical and mental energy [61] and is similar to the MBI’s definition of “feelings of exhaustion, in general” ([45], p. 40). Mental distance refers to psychological distancing from work or people at work [61], which is similar to Cynicism/Depersonalisation in the MBI, defined as “feelings of indifference or a distant attitude towards work” ([45], p. 40). Emotional and Cognitive impairment is unique to the BAT. The former is emotional reactions, such as frustration and irritability, brought on by burnout; the latter is “memory problems, attention and concentration deficits and poor cognitive performance” ([61], p. 27).

### The dimensionality of the BAT

Dimensionality refers to the number of latent factors summarising the common variance in item responses [10]. The BAT has four theoretical dimensions. Research supports these four dimensions. For example, Schaufeli et al. [61] found four components in the BAT item correlation matrix using the eigenvalue greater than one and scree test criteria, and Consiglio et al. [14] found four components with eigenvalues greater than one. Some studies compare the fit of alternative models, such as a correlated factor model and second-order model, to a one-factor model. Schaufeli et al. [61, 62] found that these two models fit better than a one-factor model. Other studies concluded that a second-order model fits best (e.g., [4, 30, 48, 72]). A second-order model implies that the four BAT dimensions are correlated and that a higher-order general burnout factor explains these correlations (e.g., [11]). In this model, the group factors mediate the relationship between the second-order factor and items because the items do not directly load on the general factor. In contrast, a bifactor model places the general and group factors on the same hierarchical level. Therefore, it decomposes item variance between general and group factors instead of through group factors [11].

We know of two studies that applied a bifactor model to the BAT items. Sakakibara et al. [58] found that the general factor accounted for 70% of the common variance in their sample from Japan. Inspection of their pattern matrix shows that item EX2 had a negligible general factor loading, with only 36% of the item common variance explained by the general factor. The Mental Distance, Cognitive, and Emotional Impairment items were also almost entirely dominated by the general factor. De Beer et al. [16] found that a bifactor model best fit the data for their South African sample. They do not report the percentage of common variance the general factor explains. However, using their pattern matrix, we calculated that this factor accounted for 64% of the common variance. As with Sakakibara et al. [58], item EX2 had a negligible factor loading. These two studies suggest that the general factor explains most item variance.

### Rasch model applied to the BAT

The Rasch model is a probabilistic model that indicates if items adhere to fundamental measurement properties. These properties mean that items can be summed [8]. Hadžibajramović et al. [31] applied the unidimensional Rasch model to the BAT items with data from the Netherlands and Flanders. Regarding dimensionality, principal components analysis of the standardised Rasch residuals and the residual correlation matrices showed evidence for more than one dimension in the item responses, implying that there are possibly multiple dimensions. Their result supported the BAT’s five-point response scale and showed that most items fit the model. Only three items, EX7, MD2, and EI3, did not adequately fit the model. Sinval et al. [65] used the multidimensional Rasch model with data from Brazil and Portugal. Most items, except for EX2, MD2, MD5, and CI2, fit the model. Both studies also found that the Exhaustion and Mental Distance items required less of the trait to endorse than the Emotional and Cognitive Impairment items.

### The convergent validity of the BAT and MBI scale scores

Correlation coefficient matrices in existing studies show that the BAT Exhaustion and Mental Distance scale scores have the strongest linear relationships with the MBI Exhaustion and Cynicism scale scores ([4, 48, 58]). Studies using the multitrait-multimethod (MTMM) approach have concluded that the BAT and MBI have convergent validity. However, this conclusion must be cautiously treated because it is based on nested models. The BAT and MBI method factors typically correlate above 0.80 ([14, 16, 58, 61]), which suggests that they share a large proportion of method and trait variance (e.g., [44]) with little unique variance at the factor level [61].

### Measurement invariance of the BAT

Research suggests that burnout is a typical response to demands and resources regardless of the country, but that cross-cultural differences exist in the experience and possible antecedents of burnout (e.g., [6, 46, 50, 59]). In this regard, Rattrie et al. [52] found that the relationship between workplace demands and burnout differed across some of Hofstede’s cultural dimensions. Similarly, Fish et al. [24] suggest that cultural differences, such as individualism or collectivism, might explain the experience and consequences of burnout. Commenting on cross-country differences, Schaufeli [60] writes that “even within Western culture ‘burnout’ can mean different things in different countries, ranging from mild psychological distress to a medically diagnosed incapacity to work” (p. 124).

Cultural differences can introduce irrelevant variance or bias from a measurement perspective, rendering score comparisons meaningless [66] unless research shows measurement invariance for a particular measure. Research supports the measurement invariance of the BAT second-order model across different cultural groups [17, 65]. However, as previously mentioned, these studies focused on the model, whereas we are interested in bias in the items.

## Summary

The BAT is a new burnout measure that has shown good psychometric properties. However, some unresolved questions remain. These questions are (a) the appropriate level of score interpretation, (b) its convergent validity with the MBI, and (c) measurement invariance in countries outside of Europe.

## Method

### Participants

We obtained 800 responses to the BAT and MBI between 2020 and 2022 using non-probability sampling. We removed six participants who failed the attention check questions or did not consent for us to use their data (see the Online Supplement for more information). The final sample consisted of 794 participants from Australia (*n* = 200), the Netherlands (*n* = 199), South Africa (*n* = 197), and the United States (*n* = 198). The mean age of the participants was 30.09 (median = 28, standard deviation = 9.47) and ranged from 18 to 72 years. Most of the participants identified as a woman (*n* = 497, 62.60%), followed by man (*n* = 276, 34.80%) and non-binary (*n* = 16, 1.90%) identification. The participants generally had full-time (*n* = 502, 63.20%) or part-time (*n* = 259, 32.60%) employment. Employed participants indicated that they worked from both their home and office (*n* = 288, 36.30%), the office exclusively (*n* = 36.30%), home exclusively (*n* = 154, 19.40%), or had some other work arrangement (*n* = 38, 4.80%). The participants worked for 34 h on average (median = 38, standard deviation = 12.79) and approximately 4.53 days (median = 5, standard deviation = 1.17) per week.

### Procedure

We placed the questionnaires on Google Forms and used Prolific—an online research platform—to obtain participants. The inclusion criteria to participate were that participants resided in one of the four countries, were older than 18 years, and currently worked or had had at least one job. Participants were invited via Prolific to complete a biographical information section, the BAT, and the MBI. The participants were paid approximately £2.60 (3$) for completing the questionnaires. We included four attention-check questions to screen out potentially invalid responses.

### Instruments

Participants completed the 23-item BAT and the 16-item MBI General Survey. The MBI consists of three scales: Exhaustion, Cynicism, and Professional Efficacy. Studies support the psychometric properties of the MBI in Australia [29, 38], the Netherlands [5, 64], South Africa [18, 69], and the United States [7, 25]. We included three single-item questions in the biographic questionnaire to measure job complexity, satisfaction, and turnover intentions. The job complexity and satisfaction items had a 1 to 10 rating scale from 1 = *not at all complex* to 10 = *very complex* and 1 = *not at all satisfied* to 10 = *very satisfied*. Lastly, we used a dichotomous *yes* or *no* response format to measure turnover intentions.

### Data analysis

#### Correlation coefficients and reliability coefficients

We used Pearson and distance correlation coefficients to investigate the linear and non-linear relationships between the BAT and MBI scale scores using the *Hmisc* version 4.6-0 [34] and *energy* version 1.7-9 [54] packages in *R* version 4.1.2 [51]. We also calculated the multiple correlation coefficient with each respective BAT or MBI scale as the dependent variable and the MBI or BAT scales as the independent variables. Cronbach’s alpha and omega total reliability coefficients were calculated using the *MBESS* version 4.9.0 [36] package.

### Dimensionality and convergent validity

We investigated dimensionality using the empirical Kaiser criterion, parallel analysis of components, and the hull method in the *EFAtools* package version 0.4.0 [68]. We added resampling to determine consistency [27]. Several options are available for analysing a bifactor structure. An important consideration is a confirmatory or exploratory estimation (see [26, 53]). Confirmatory models typically constrain factor loadings so that an item has two non-zero loadings, one on the general factor and one on the group factor. Cross-loadings are constrained to zero. Exploratory models relax the constraint on cross-loadings so that a general factor and multiple group factors influence items [53].

We used an exploratory orthogonal target-rotated bifactor model with a pre-specified target matrix for three reasons. First, exploratory models allow researchers to identify inadequate model fit directly. Second, forcing zero cross-loadings can lead to biased factor loading estimates [53]. Third, we were interested in examining the cross-loadings between the BAT and MBI to understand better how the scales converge. We estimated the model using unweighted least square estimation in the *EFAutilities* package version 2.1.1 [76] using polychoric correlation for the BAT Combined Sample and Pearson correlation coefficients for the country samples and the combined BAT and MBI items. We opted for Pearson correlation coefficients to prevent biased estimation due to small sample sizes or too many empty cells in the contingency tables (see [42]).

We investigated the factor sizes and replicability using the explained common variance (ECV), coefficient omega hierarchical and subscale, relative omega, and coefficient *H.* The ECV is the proportion of common variance in each factor, with a general factor ECV > 0.70 or 0.80 indicating essential unidimensionality [55]. Coefficient omega hierarchical is the proportion of reliable variance in each factor. Relative omega is the ratio of coefficient omega hierarchical to coefficient omega total, which indicates the proportion of reliable variance in each factor after controlling for the other factors [20, 55]. Coefficient *H* indicates the construct replicability of each factor, with a value > 0.80 indicating replicability [33, 55]. We then calculated the item-explained common variance (I-ECV) and absolute relative bias. The former indicates the proportion of variance in each item explained by the general factor, and the latter indicates the difference between factor loadings from a one-factor model and the general factor [70]. An absolute relative bias > 10–15% indicates potential multidimensionality [55]. However, this bias is small when the ECV and percentage uncontaminated correlation coefficients (PUC) are > 0.70^1^ [56]. We used the *BifactorIndicesCalculator* package version 0.2.2 [19] to calculate the bifactor indices.

We investigated the added value of multiple factor score estimates using Ferrando and Lorenzo-Seva’s ([23], p. 257, formula 18) weighted proportional reduction in mean square errors. Hereafter, we investigated dimensionality based on the incremental variance in prediction using job complexity, satisfaction, and turnover intentions as the external variables ([22], p. 444). Both analyses were conducted in the *Factor* software version 12.01.02 [41] with unweighted least squares estimation and an oblique target rotation. Polychoric or Pearson correlation coefficients were used as input and 95% bias-corrected and accelerated confidence intervals were obtained using 1000 bootstrapped samples.

### Rasch model and differential item functioning

We investigated the fit of the BAT items to the Rasch Rating Scale model in *Winsteps* version 5.2.20 [40]. This model is a polytomous extension of the dichotomous model [3]. We first investigated the suitability of the BAT five-point response format with category threshold functioning. Then we used principal components analysis of standardised residuals [67] and Yen’s expected value-adjusted Q3 statistic [75] to investigate dimensionality. We calculated the mean eigenvalue of the first and second principal components using 1000 simulations to obtain empirical cut-off values for multidimensionality. An adjusted Q3 correlation coefficient > |0.30| indicated multidimensionality (see [13]). We investigated the fit of the items to the Rasch model with infit and outfit mean square statistics, which are chi-square statistics divided by their degrees of freedom. The expected value of these statistics is 1.00. We considered an item to have an inadequate fit when its 95% bias-corrected and accelerated confidence interval did not include 1.00. The *coxed* package version 0.3.3 [37] was used to obtain these confidence intervals based on 1000 resamples. We were only concerned with infit or outfit mean square statistics > 1.00 because this value means that an item contains unmodelled noise [74].

After fitting the Rasch model, we investigated differential item functioning (DIF) using ordinal logistic regression [15] with the *lordif* package [12] (version 0.3-3). The Rasch person measures served as the conditioning variable. This DIF approach compares three models representing a baseline (Model 1), uniform DIF (Model 2), and non-uniform DIF (Model 3). We first compared the fit of model 1 and model 3 with a likelihood-ratio test to obtain an overall DIF effect size. Hereafter, we determined if the DIF was non-uniform or uniform [12, 15]. We set statistical significance to *p* < .001 instead of *p* < .05 to account for Type-1 errors [77]. The change in Nagelkerke’s pseudo *R*^2^ across the three models indicates the DIF effect size (see [12]). The DIF effect labels of Jodoin and Gierl [35] determined the DIF size: Negligible (*R*^2^ = 0.035), moderate (*R*^2^ = 0.070), and large (*R*^2^ > 0.075). We first investigated DIF by comparing one country to the combined responses from all other countries. Hereafter, we investigated DIF for each pair-wise country comparison.

### Ethical considerations

The Department of Industrial Psychology and People Management research ethics committee at the University of Johannesburg provided ethical clearance for our study (IPPM-2020-471). Participants received information about the study’s purpose, how we planned to use their data, what information we required, and our intention to include anonymised item responses on the Open Science Foundation website. The consent asked participants to provide consent for us to include their data in the study and online. After completing the questionnaires, we again asked the participants to provide their consent if they changed their minds. Participants were still paid if they changed their consent. We provide a link to the Open Science Foundation in the Online Supplement for researchers who want to access the anonymous data.

## Results

### Descriptive statistics, reliability, and correlation coefficients

The BAT scale score descriptive statistics are available in the Online Supplement. The participants scored the highest on the BAT Exhaustion subscale (mean = 2.96) and lowest on the Emotional Impairment subscale (mean = 1.95). Coefficient alpha (mean = 0.85) and omega total (mean = 0.86) reliability coefficients were > 0.80 for all four scale scores. Table 1 presents Pearson, distance, and multiple correlation coefficients. The BAT Exhaustion and Mental Distance scale scores had the largest linear relationships with the MBI Exhaustion (*r* = .83 and *r* = .62) and Cynicism (*r* = .57 and *r* = .79) scale scores, suggesting that these scales measure similar constructs.

**Table 1:** Pearson Correlation, Distance, and Multiple Correlation Coefficients for the BAT and MBI Scale Scores

	Scale	1	2	3	4	5	6	7	8	9
1.	BAT Exhaustion	**0.83**	0.53	0.50	0.47	.	0.80	0.54	0.24	0.73
2.	Mental Distance	0.58	**0.81**	0.47	0.43	.	0.57	0.75	0.38	0.72
3.	Cognitive Impairment	0.57	0.52	**0.61**	0.43	.	0.48	0.47	0.38	0.52
4.	Emotional Impairment	0.52	0.48	0.49	**0.55**	.	0.47	0.42	0.28	0.49
5.	BAT Total	.	.	.	.	**0.85**	0.77	0.70	0.39	0.81
6.	MBI Exhaustion	0.83	0.62	0.53	0.51	0.80	**0.85**	0.63	0.24	.
7.	Cynicism	0.57	0.79	0.50	0.46	0.72	0.67	**0.80**	0.37	.
8.	Professional Efficacy	-0.26	-0.40	-0.39	-0.30	-0.41	-0.24	-0.37	**0.46**	0.32
9.	MBI Total	0.77	0.77	0.57	0.53	0.83	.	.	-0.33	**0.87**

*Note.* Pearson correlation coefficients below the diagonal and distance correlation coefficients above the diagonal. Multiple correlation coefficients on the diagonal in bold. MBI Total does not include Professional Efficacy items

### Dimensionality

Table 2 presents the recommended number of components or factors to extract from the BAT item correlation matrix. The empirical Kaiser criterion (99% consistency) and parallel analysis (100% consistency) suggested extracting four components, with the first linear combination in the item correlation matrix explaining 39.91% of the total variance. The Hull method suggested extracting five factors (58% consistency). The results for each country were less consistent, likely due to the smaller sample sizes, with one to four components or factors suggested.

**Table 2:** Recommended Number of BAT Factors or Components to Extract

Sample	EKC	%	Parallel	%	Hull	%
Combined	4	99	4	100	5	58
Australia	3	52	3	63	4	31
Netherlands	3	52	4	77	1	58
South Africa	3	62	3	78	1	30
United States	4	59	4	91	4	29

*Note*. EKC = empirical Kaiser criterion, Parallel = parallel analysis of components, Hull = Hull method. Values under % indicate consistency for each technique (1000 bootstrap samples used)

Table 3 shows statistically significant factor loadings (*p* < .001) in the bifactor pattern matrix. The pattern matrix supports a large general factor with four smaller group factors (see Table 4). The general factor explained most of the variance in the BAT Exhaustion (mean = 0.65), Mental Distance (mean = 0.61), and Cognitive Impairment (mean = 0.58) items. Seven items had I-ECV < 0.50, with item EX2 showing the lowest I-ECV and general factor loading (λ = 0.06). Results for each country were similar, except that item EX2 had a stronger general factor loading in Australia (λ = 0.31) and the Netherlands (λ = 0.36).

**Table 3:** Target Rotated Bifactor Model and Item Explained Common Variance

Item	General	Exhaustion	Mental	Cognitive	Emotional	I-ECV
EX1	0.65	0.47	.	.	.	**0.65**
EX2	.	0.69	.	.	0.12	0.01
EX3	0.67	0.43	.	.	.	**0.71**
EX4	0.54	0.40	.	-0.19	0.10	**0.59**
EX5	0.75	.	.	.	-0.11	**0.93**
EX6	0.72	0.24	.	0.14	.	**0.87**
EX7	0.68	0.38	.	.	.	**0.75**
EX8	0.70	0.48	.	.	.	**0.67**
MD1	0.85	.	0.26	.	-0.18	**0.88**
MD2	0.57	.	.	.	.	**0.85**
MD3	0.49	.	0.64	.	0.12	0.34
MD4	0.59	-0.13	0.53	.	.	**0.55**
MD5	0.48	.	0.49	0.13	.	0.45
CI1	0.67	.	.	0.53	-0.08	**0.61**
CI2	0.68	0.10	.	0.54	.	**0.60**
CI3	0.67	.	.	0.60	.	**0.55**
CI4	0.69	.	.	0.65	.	**0.53**
CI5	0.55	.	.	0.41	0.21	**0.59**
EI1	0.55	.	.	.	0.61	0.44
EI2	0.53	.	.	.	0.60	0.43
EI3	0.46	.	.	.	0.47	0.47
EI4	0.66	.	.	.	0.43	**0.69**
EI5	0.45	.	.	.	0.70	0.29

*Note*. EX = BAT Exhaustion, MD = Mental Distance, CI = Cognitive Impairment, EI = Emotional Impairment, General = General Factor, Exhaustion = BAT Exhaustion, Mental = Mental Distance, Cognitive = Cognitive Impairment, Emotional = Emotional Impairment, I-ECV = item explained common variance. Shown factor loadings are statistically significant at *p* < .001. Item explained common variance > 0.50 are given in bold

**Table 4:** Bifactor Indices for the Target Rotated Bifactor Models

Sample	Index	General Factor	Exhaustion	Mental	Cognitive	Emotional
Combined	ECV	0.59	0.11	0.07	0.11	0.12
	ω_h_	0.82	0.29	0.28	0.38	0.47
	ω_Rel_.	0.85	0.30	0.29	0.40	0.48
	H	0.94	0.69	0.60	0.69	0.72
Australia	ECV	0.62	0.11	0.06	0.12	0.09
	ω_h_	0.85	0.28	0.22	0.38	0.34
	ω_Rel_.	0.88	0.29	0.23	0.40	0.36
	H	0.94	0.65	0.53	0.70	0.62
Netherlands	ECV	0.60	0.09	0.09	0.12	0.10
	ω_h_	0.85	0.14	0.33	0.39	0.41
	ω_Rel_.	0.89	0.14	0.35	0.41	0.43
	H	0.93	0.51	0.57	0.68	0.66
South Africa	ECV	0.60	0.12	0.06	0.11	0.12
	ω_h_	0.81	0.30	0.14	0.34	0.48
	ω_Rel_.	0.85	0.31	0.15	0.35	0.51
	H	0.94	0.64	0.46	0.63	0.67
United States	ECV	0.49	0.14	0.10	0.14	0.14
	ω_h_	0.75	0.34	0.39	0.48	0.53
	ω_Rel_.	0.79	0.36	0.41	0.51	0.56
	H	0.91	0.71	0.65	0.74	0.74

*Note.* EX = BAT Exhaustion, MD = Mental Distance, CI = Cognitive Impairment, EI = Emotional Impairment, General = General Factor, Exhaustion = BAT Exhaustion, Mental = Mental Distance, Cognitive = Cognitive Impairment, Emotional = Emotional Impairment. ECV = explained common variance, ω_h_ = coefficient omega hierarchical, ω_Rel_. = relative omega, H = construct replicability

Table 4 shows the bifactor model indices. The general factor explained 59% of the common variance. The group factors' explained variance ranged from 7 to 12%. Coefficients omega hierarchical and relative were 0.82 and 0.85, respectively. The general factor was the only well-defined factor with a coefficient *H* of 0.94. We found similar results to those reported above for each country. One noticeable difference is the explained common variance of the general factor and coefficient omega hierarchical of 0.49 and 0.75 in the United States. There was a 22% bias between the combined sample’s unidimensional and general factor loading. However, it appeared that item EX2 was mostly responsible for this bias because of its small general factor loading. The bias was 10% after removing this item. Similar results occurred for each country. In the United States sample, removing item EX2 reduced the mean bias from 51 to 10%. The proportional reduction in mean square errors suggested multidimensionality, with the group factors showing incremental validity in the prediction of Satisfaction (*r*_difference_ = 0.50) and Complexity (*r*_difference_ = 0.40) but not Turnover (*r*_difference_ = -0.01). The Online Supplement provides more detailed information on these results.

### Convergent validity

As previously mentioned, we use the term convergent and discriminant validity to mean the extent to which the BAT and MBI items load on general or group factors. Before running a bifactor model, we combined the two questionnaires to investigate how many components or factors best describe their combined structure. The two questionnaires should have seven dimensions if they are not measuring something in common. The empirical Kaiser criterion (100% consistency) and parallel analysis (89% consistency) recommended extracting five components, with the first linear combination in the item correlation matrix explaining 37.45% of the total variance. Therefore, the two questionnaires appear to have some similarities. The Hull method suggested extracting one factor, but the consistency was only 38%. We found similar results for each country.

We then proceeded with a bifactor model with the target matrix specifying one general factor and seven group factors. The Professional Efficacy items were reverse-scored for this analysis to remove the negative sign on the general factor. Table 5 presents the bifactor pattern matrix’s statistically significant factor loadings (*p* < .001). Bifactor indices are available from the Online Supplement. The results support the presence of a general factor with five instead of seven group factors. The BAT and MBI Exhaustion items formed one factor, and the general factor completely dominated the BAT Mental Distance and MBI Cynicism items. The Cognitive Impairment, Emotional Impairment, and Professional Efficacy items had the most well-defined group factors. We found similar results to those reported above for each country.

**Table 5:** *Target Rotated Bifactor Model and Item Explained Common Variance for the BAT and MBI Items*

	G	BEX	MD	CI	EI	MEX	CY	PE	I-ECV
BEX1	0.63	0.34	.	.	.	.	.	.	**0.68**
BEX2	0.16	0.57	.	.	.	.	.	.	0.06
BEX3	0.65	0.28	.	.	.	0.25	.	.	**0.74**
BEX4	0.54	0.37	.	-0.14	.	.	.	.	**0.59**
BEX5	0.71	.	.	.	.	.	.	.	**0.82**
BEX6	0.68	0.22	.	0.18	.	.	.	.	**0.84**
BEX7	0.67	0.35	.	.	.	.	.	.	**0.73**
BEX8	0.66	0.33	.	.	.	0.32	.	.	**0.66**
MD1	0.80	.	.	.	-0.10	.	.	.	**0.82**
MD2	0.51	.	.	.	.	.	.	.	**0.79**
MD3	0.60	.	.	-0.19	.	.	.	.	**0.71**
MD4	0.63	.	.	.	.	.	.	.	**0.78**
MD5	0.61	.	0.36	.	.	.	.	.	**0.66**
CI1	0.57	.	.	0.58	.	.	.	.	0.48
CI2	0.60	.	.	0.56	.	.	.	.	**0.52**
CI3	0.58	.	.	0.61	.	.	.	.	0.47
CI4	0.59	.	.	0.68	.	.	.	.	0.43
CI5	0.46	.	.	0.42	0.22	.	.	.	0.46
EI1	0.45	.	.	.	0.62	.	.	.	0.34
EI2	0.44	.	.	.	0.56	.	.	0.11	0.37
EI3	0.44	.	.	.	0.43	.	.	.	**0.50**
EI4	0.60	.	.	.	0.40	.	.	.	**0.68**
EI5	0.40	.	.	.	0.66	.	.	.	0.26
MEX1	0.74	0.19	.	.	.	0.46	.	-0.08	**0.67**
MEX2	0.71	0.20	.	.	.	0.44	.	-0.12	**0.67**
MEX3	0.79	.	.	.	.	0.30	.	.	**0.84**
MEX4	0.75	0.17	.	.	.	0.29	.	.	**0.82**
MEX5	0.75	0.21	.	.	.	0.36	.	-0.10	**0.74**
CY1	0.75	.	.	.	.	.	.	.	**0.68**
CY2	0.77	.	.	.	-0.10	.	.	.	**0.70**
CY3	0.43	.	.	.	.	.	.	-0.25	**0.68**
CY4	0.71	.	0.45	.	.	.	.	.	**0.65**
CY5	0.68	-0.18	.	.	.	.	.	0.19	**0.69**
PE1	.	0.15	.	.	0.17	.	.	0.52	0.03
PE2	0.40	.	.	.	.	.	.	0.58	0.30
PE3	0.18	0.13	.	0.17	.	.	.	0.67	0.06
PE4	0.38	-0.31	.	.	.	.	.	0.45	0.31
PE5	0.44	-0.31	.	.	.	.	.	0.57	0.30
PE6	0.25	0.16	.	0.22	.	.	.	0.69	0.10

*Note*. BEX = BAT Exhaustion, MD = Mental Distance, CI = Cognitive Impairment, EI = Emotional Impairment, MEX = MBI Exhaustion, CY = Cynicism, PE = Professional Efficacy, G = General Factor, I-ECV = item-explained common variance. Shown factor loadings are statistically significant at *p* < .001. Item explained common variance ≥ 0.50 are given in bold

### Rasch rating scale model

#### Category thresholds

Table 6 presents the response category summary statistics. The observed average logits (minimum = -2.10, maximum = 1.27), indicating the average person measure in each response category, and Rasch-Andrich thresholds (minimum = -2.13, maximum = 1.94), indicating the trait coverage of the response options, increased monotonically. There was also no unsatisfactory fit to the model for each category. The results were similar for each country.

**Table 6:** Item category response function

	Observed	Expected	Infit	Outfit	Andrich
Never	-2.10	-2.03	0.95	0.96	.
Rarely	-1.06	-1.11	1.04	0.99	-2.13
Sometimes	-0.33	-0.33	0.96	1.00	-0.61
Often	0.36	0.38	1.02	1.03	0.79
Always	1.27	1.29	1.08	1.14	1.94

*Note.* Observed = average observed logit, Expected = average model expected logit, Andrich = Rasch-Andrich Thresholds

### Item locations and fit statistics

Table 7 presents the Rasch item location and item fit statistics, with a Wright map of these locations available in the Online Supplement. The item locations ranged from -1.21 to 1.42. We calculated the mean of the item logits for each scale, which showed that the Exhaustion (mean = -0.71) and Mental Distance (mean = -0.05) items were the easiest to endorse, and the Cognitive Impairment (mean = 0.20) and Emotional Impairment (mean = 0.99) items the most difficult items to endorse. Items EX2, MD2, MD3, and MD5 had a poor fit to the Rasch model. We provide item characteristic curves for these items in the Online Supplement. Item EX2 consistently showed the worst fit with an infit and outfit mean square of 1.63 and 1.78 in the combined sample. It’s fit to the model was particularly poor in South Africa, with an infit and outfit mean square of 2.04 and 2.29. The Mental Distance items also consistently showed poor fit across the four countries.

**Table 7:** Rasch Item Locations and Fit Statistics

Item	Location	SE	Infit	Z_Infit_	Outfit	Z_Outfit_
EX1	-0.84	0.04	0.63 [0.53, 0.75]	-8.96	0.68 [0.55, 0.91]	-7.57
EX2	-1.21	0.04	**1.63 [1.40, 1.89]**	9.90	**1.78 [1.46, 2.20]**	9.90
EX3	-0.80	0.04	0.85 [0.73, 0.98]	-3.29	0.84 [0.72, 0.96]	-3.41
EX4	-0.40	0.04	1.05 [0.88, 1.21]	0.99	1.04 [0.88, 1.19]	0.79
EX5	-0.85	0.04	0.89 [0.75, 1.05]	-2.47	0.89 [0.75, 1.04]	-2.36
EX6	-0.16	0.04	0.81 [0.67, 0.94]	-4.30	0.80 [0.66, 0.93]	-4.52
EX7	-0.31	0.04	0.79 [0.69, 0.90]	-4.79	0.78 [0.67, 0.89]	-4.97
EX8	-1.14	0.04	0.81 [0.70, 0.93]	-4.37	0.80 [0.70, 0.92]	-4.45
MD1	-0.46	0.04	0.90 [0.79, 1.05]	-2.22	0.89 [0.78, 1.03]	-2.27
MD2	-0.27	0.04	**1.37 [1.20, 1.54]**	6.84	**1.39 [1.23, 1.59]**	7.26
MD3	0.27	0.05	**1.34 [1.14, 1.57]**	6.26	**1.39 [1.16, 1.65]**	7.05
MD4	0.14	0.05	1.13 [0.97, 1.33]	2.50	1.13 [0.98, 1.34]	2.66
MD5	0.06	0.05	**1.38 [1.18, 1.56]**	6.98	**1.42 [1.21, 1.65]**	7.59
CI1	-0.21	0.04	0.88 [0.79, 1.02]	-2.47	0.87 [0.77, 0.98]	-2.82
CI2	0.19	0.05	0.69 [0.61, 0.80]	-7.10	0.68 [0.61, 0.77]	-7.27
CI3	0.33	0.05	0.78 [0.66, 0.91]	-4.96	0.76 [0.67, 0.89]	-5.21
CI4	0.14	0.05	0.77 [0.70, 0.88]	-5.00	0.76 [0.69, 0.87]	-5.23
CI5	0.55	0.05	0.78 [0.67, 0.9]	-4.70	0.81 [0.68, 0.93]	-4.05
EI1	1.25	0.05	1.03 [0.89, 1.25]	0.66	0.98 [0.84, 1.16]	-0.31
EI2	1.42	0.05	1.28 [1.08, 1.49]	4.90	1.18 [0.98, 1.39]	2.93
EI3	0.15	0.05	1.18 [0.99, 1.36]	3.58	1.18 [1.00, 1.37]	3.51
EI4	1.04	0.05	1.05 [0.91, 1.21]	0.93	0.97 [0.85, 1.11]	-0.58
EI5	1.09	0.05	1.22 [1.03, 1.38]	4.06	1.19 [1.00, 1.39]	3.27

*Note.* EX = BAT Exhaustion, MD = Mental Distance, CI = Cognitive Impairment, EI = Emotional Impairment, SE = standard error of the location. 95% bias-corrected and accelerated confidence intervals in parentheses (1000 samples). Infit and outfit mean squares showing underfit in bold

### Rasch dimensionality

The first component of the standardised residual matrix had an eigenvalue of 3.25 and explained 14.10% of the residual variance. This eigenvalue is larger than the simulated eigenvalue of 1.36 from data that fit the model. This component contrasted the Exhaustion items with the Cognitive Impairment items. These contrasting items had a disattenuated correlation coefficient of *r* = .63. The second component had an eigenvalue of 2.79 and explained 12.10% of the residual variance. As with the first component, this eigenvalue was larger than the simulated eigenvalue of 1.30. The disattenuated correlation coefficient between the contrasting items on this component was *r* = .68. Yen’s Q3 coefficient showed non-negligible local dependence in 41 (16%) item pairs. The Cognitive (mean = 0.37) and Emotional Impairment (mean = 0.25) items had the most local dependence.

### Differential item functioning

After fitting the unidimensional Rasch model, we investigated DIF using the Rasch person measures as the trait score. Table 8 presents the χ2 difference test *p* values and change in Nagelkerke *R*^2^ between Model 1 (no DIF) and Model 3 (total DIF). The full results are in the Online Supplement. Ten items, two in the Netherlands, seven in South Africa, and one in the United States, had statistically significant DIF. These items are EX2 (twice), EX3, MD3, CI1, CI2, CI3, CI4 (twice), and EI3. All of these items showed uniform rather than non-uniform DIF. However, only items EX2 and MD11 in South Africa had non-negligible DIF effect sizes. There was little DIF between Australia, the Netherlands, and the United States. Regarding South Africa, the following items showed statistically significant DIF: Australia and the United States = EX2 and MD3, the Netherlands = EX2, MD3, CI1, and CI4. Items EX2 and MD3 had the largest DIF effect size.

**Table 8:** Total differential item functioning Model 1 versus Model 3

	*χ*^*2*^ difference test *p* values				Change in Nagelkerke *R*^2^			
Item	Australia	Netherlands	South Africa	United States	Australia	Netherlands	South Africa	United States
EX1	0.080	0.018	0.734	0.191	0.00	0.01	0.00	0.00
EX2	0.040	**0.000**	**0.000**	0.350	0.01	0.02	**0.08**	0.00
EX3	0.045	0.107	**0.000**	0.746	0.00	0.00	0.01	0.00
EX4	0.605	0.120	0.059	0.543	0.00	0.00	0.01	0.00
EX5	0.240	**0.004**	0.298	0.846	0.00	0.01	0.00	0.00
EX6	0.840	0.747	0.668	0.573	0.00	0.00	0.00	0.00
EX7	0.991	0.467	0.210	0.081	0.00	0.00	0.00	0.00
EX8	0.239	0.681	0.914	0.472	0.00	0.00	0.00	0.00
MD1	0.307	0.875	0.016	0.480	0.00	0.00	0.01	0.00
MD2	0.226	0.750	**0.004**	0.094	0.00	0.00	0.01	0.01
MD3	0.128	**0.003**	**0.000**	0.058	0.00	0.01	**0.04**	0.01
MD4	0.318	0.040	0.963	0.438	0.00	0.01	0.00	0.00
MD5	0.897	0.050	0.005	0.467	0.00	0.01	0.01	0.00
CI1	0.184	**0.002**	**0.000**	0.872	0.00	0.01	0.02	0.00
CI2	0.775	0.006	**0.000**	0.192	0.00	0.01	0.01	0.00
CI3	0.814	0.010	**0.000**	0.794	0.00	0.01	0.01	0.00
CI4	0.645	**0.000**	**0.000**	0.885	0.00	0.01	0.02	0.00
CI5	0.435	0.594	0.204	0.922	0.00	0.00	0.00	0.00
EI1	0.977	0.648	0.867	0.429	0.00	0.00	0.00	0.00
EI2	0.301	0.979	0.534	0.620	0.00	0.00	0.00	0.00
EI3	0.183	0.025	0.033	**0.001**	0.00	0.01	0.01	0.01
EI4	0.065	0.705	0.530	**0.004**	0.00	0.00	0.00	0.01
EI5	0.229	0.591	0.703	0.900	0.00	0.00	0.00	0.00

*Note.* EX = Exhaustion, MD = Mental Distance, CI = Cognitive Impairment, EI = Emotional Impairment. *p* values < 0.005 and *R*^2^ change values > 0.035 are in bold

## Discussion

Our study investigated (a) the appropriate level of score interpretation of the BAT, (b) its convergent validity with the MBI from the perspective of bifactor modelling, and (c) cross-country differential item functioning. We discuss the results for these questions below and then present some implications and recommendations for using the BAT.

### Appropriate level of score interpretation

The BAT has two scoring options: a general (or total) burnout score or four dimension scores [61]. We first investigated the major number of dimensions. Similar to Schaufeli et al. [61] and Consiglio et al. [14], our results support four major dimensions. However, the large inter-scale correlation coefficients suggest a general factor. The bifactor model showed that the general factor accounted for more than half of the common and reliable variance and explained more variance in the Exhaustion and Mental Distance than the Cognitive and Emotional Impairment items. This result suggests that the two latter scales have unique variance not shared with the other scales. Results from Hadžibajramović et al. [31] and our Rasch analysis support this suggestion. The proportional reduction in mean-squared error and external variable analyses also suggested that group factors are warranted.

Regarding the appropriate level of score interpretation, our results, combined with those from Sakakibara et al. [58] and de Beer et al. [16], suggest calculating and interpreting a general burnout score. However, it might be useful to calculate group scores, particularly for Cognitive and Emotional Impairment, if a more nuanced understanding of the general burnout score is desired (see [61]). That said, the reliabilities of the group scores after controlling for the general factor will likely be too low to be used for decision-making purposes. Therefore, it is probably better to use group scores as additional information to help explain general burnout scores. Further research is needed to determine if our suggestion is viable (see [20]).

### The convergent validity of the BAT with the MBI

The second question we investigated is the convergent and discriminant validity of the BAT and MBI. Studies show that the BAT and MBI measure the same general burnout construct, but some scales diverge [14, 16, 58, 61]. We first investigated the number of major dimensions in the combined BAT and MBI responses. The results showed five major dimensions, which suggests that the two measures share common constructs. We then used bifactor modelling to investigate this similarity. The bifactor model showed a prominent general factor, explaining the large correlation de Beer et al. [16] found between the BAT and MBI general factors. These results indicate that the BAT and MBI measure the same burnout construct. They also show that the BAT and MBI Exhaustion items and the Mental Distance and Cynicism items converge at the group level. The BAT and MBI Cognitive Impairment, Emotional Impairment, and Professional Efficacy items diverge. However, the general factor loadings of the Professional Efficacy items suggest that the scale is not part of burnout [17, 61]. Together, our results show that the BAT and MBI general burnout, Exhaustion, Mental Distance, and Cynicism scales converge, and the Emotional and Cognitive Impairment scales are unique to the BAT. Therefore, the BAT is a more comprehensive burnout measure.

### Rasch rating scale model and differential item functioning

The third question we investigated is the differential item functioning (DIF) of the BAT items. We first fit the items to the Rasch model. The results supported using a five-point scale, showing that the BAT can measure people low and high on burnout. As with Hadžibajramović et al. [31] and Sinval et al. [65], the Exhaustion and Mental Distance items provide most information on the lower end of burnout and the Emotional and Cognitive Impairment items at the upper end. Most items fit the Rasch model, meaning a general burnout score can be made by summing them. However, items EX2, MD2, and MD5—the same items identified as potentially problematic in the studies above—had inadequate fit to the model. Item EX2 consistently showed inadequate fit, which leads us to question its continued use in the BAT. Few items showed DIF, which agrees with the measurement invariance found by de Beer et al. [17]. The items had only uniform DIF, implying they were not on the same measurement scale. Of the DIF items, only EX2 and MD3 showed non-negligible effect sizes. Most of the DIF comes from the South African data. It is difficult to determine what might cause conditional differences in item responses and whether statistical DIF equates to bias [66]. Possible reasons related to South Africa could be stigma, social acceptance, or limited information about psychological conditions such as burnout [2, 32, 47], which might influence how the participants responded to the items. Another potential explanation is that the BAT item descriptions might differ from how people describe burnout in other countries [71]. Therefore, although the latent burnout trait is the same in different countries, how people interpret the item descriptions might differ, leading to conditional response differences. However, we caution against speculating until other studies reproduce our results, lest the DIF does not generalise outside our sample.

### Limitations

Our study has several limitations that necessitate caution in interpreting the results. First, we only collected approximately 200 participants from each country. Fewer participants can increase the uncertainty of an estimated parameter. This uncertainty can cause problems with statistical significance tests. Smaller sample sizes are also more likely to differ from the population. Sample-specific peculiarities can limit the generalisation of the results. Together, the sample sizes can reduce the certainty of the results. Second, not all participants spoke English as their home language, which means that language rather than country might explain the DIF. Language differences might also explain the worse fit of the items to the Rasch model in South Africa (e.g., [17]). Given these limitations, we encourage readers to consider results in our study that converge with those from other studies instead of viewing our results in isolation.

## Conclusion

Our study set out to investigate the psychometric properties and differential item functioning of the BAT in Australia, the Netherlands, South Africa, and the United States. Our results support using BAT scores in these countries and the argument that the BAT is a more comprehensive measure of burnout than the MBI.

## Electronic supplementary material

Below is the link to the electronic supplementary material.

Supplementary Material 1

## Data Availability

The data and R script used in this study can be obtained from the Open Science Foundation (https://osf.io/gsaq2/?view_only=c0ed2f84c2cc4b3690b97a7fe333111c).

## Footnotes

1 The PUC for the 23 BAT items and 39 BAT and MBI items are 0.77 and 0.87.

## References

[1] Allen MJ, Yen WM (1979). Introduction to measurement theory.

[2] Anderson K. (2019). *The experience of burnout among psychologists in South Africa* [Master’s thesis, University of KwaZulu-Natal]. https://ukzn-dspace.ukzn.ac.za/handle/10413/19968

[3] Andrich DA (1978). Rating formulation for ordered response categories. Psychometrika.

[4] Angelini G, Buonomo I, Benevene P, Consiglio P, Romano L, Fiorilli C (2021). The Burnout Assessment Tool (BAT): a contribution to italian validation with teachers’. Sustainability.

[5] Bakker AB, Demerouti E, Schaufeli WB (2002). Validation of the Maslach Burnout Inventory-General Survey: an internet study. Anxiety Stress Coping.

[6] Barker GG, Volk F, Peters C (2021). Cultural influences on burnout: a swedish–american comparison. Int J Workplace Health Manage.

[7] Bebiroglu N, Bayot M, Brion B, Denis L, Pirsoul T, Roskam I, Mikolajczak M (2021). An instrument to operationalize the balance between risks and resources and predict job burnout. Int J Environ Res Public Health.

[8] Bond T, Yan Z, Heene M. Applying the Rasch model: fundamental measurement in the human sciences. Routledge; 2020.

[9] Bornovalova MA, Choate AM, Fatimah H, Petersen KJ, Wiernik BM (2020). Appropriate use of bifactor analysis in psychopathology research: appreciating benefits and limitations. Biol Psychiatry.

[10] Brown TA (2015). Confirmatory factor analysis for applied research.

[11] Chen FF, West SG, Sousa KH (2006). A comparison of bifactor and second-order models of quality of life. Multivar Behav Res.

[12] Choi SW, Gibbons LE, Crane PK (2011). lordif: an R Package for Detecting Differential Item Functioning using iterative hybrid Ordinal Logistic Regression/Item response theory and Monte Carlo Simulations. J Stat Softw.

[13] Christensen KB, Makransky G, Horton M (2017). Critical values for yen’s Q3: identification of local dependence in the Rasch model using residual correlations. Appl Psychol Meas.

[14] Consiglio C, Mazzetti G, Schaufeli WB (2021). Psychometric properties of the italian version of the Burnout Assessment Tool (BAT). Int J Environ Res Public Health.

[15] Crane PK, Gibbons LE, Jolley L, van Belle G (2006). Differential item functioning analysis with ordinal logistic regression techniques: DIF detect and difwithpar. Med Care.

[16] De Beer LT, Schaufeli WB, De Witte H (2022). The psychometric properties and measurement invariance of the Burnout Assessment Tool (BAT-23) in South Africa. BMC Public Health.

[17] De Beer LT, Schaufeli WB, De Witte H, Hakanen JJ, Shimazu A, Glaser J, Seubert C, Bosak J, Sinval J, Rudnev M (2020). Measurement invariance of the Burnout Assessment Tool (BAT) across seven cross-national representative samples. Int J Environ Res Public Health.

[18] De Vine J, Morgan B (2020). The relationship between personality facets and burnout. SA J Industrial Psychol.

[19] Dueber D. (2021). BifactorIndicesCalculator: Bifactor Indices Calculator (R package version 0.2.2) [Computer software]. https://CRAN.R-project.org/package=BifactorIndicesCalculator

[20] Dueber DM, Toland MD. (2021). A bifactor approach to subscore assessment. *Psychological Methods* Advance online publication. 10.1037/met0000459 10.1037/met0000459 34941326

[21] Eid M, Koch T, Geiser C. Multitrait–multimethod models. In: Hoyle RH, editor. Handbook of structural equation modeling. 2nd ed. Routledge; 2022. pp. 349–66.

[22] Ferrando PJ, Lorenzo-Seva U (2019). An external validity approach for assessing essential unidimensionality in correlated-factor models. Educ Psychol Meas.

[23] Ferrando PJ, Lorenzo-Seva U (2019). On the added value of multiple factor score estimates in essentially unidimensional models. Educ Psychol Meas.

[24] Fish JA, Sharplin G, Wang L, An Y, Fan X, Eckert M (2022). Cross-cultural differences in nurse burnout and the relationship with patient safety: an East-West comparative study. J Adv Nurs.

[25] Garcia HA, McGeary CA, Finley EP, Ketchum NS, McGeary DD, Peterson AL (2015). Burnout among psychiatrists in the Veterans Health Administration. Burnout Res.

[26] Giordano C, Waller NG (2020). Recovering bifactor models: a comparison of seven methods. Psychol Methods.

[27] Goretzko D, Bühner M (2022). Robustness of factor solutions in exploratory factor analysis. Behaviormetrika.

[28] Greiff S, Scherer R (2018). Still comparing apples with oranges? Some thoughts on the principles and practices of measurement invariance testing [Editorial]. Eur J Psychol Assess.

[29] Guo YF, Plummer V, Lam L, Wang Y, Cross W, Zhang JP (2019). The effects of resilience and turnover intention on nurses’ burnout: findings from a comparative cross-sectional study. J Clin Nurs.

[30] Haar J. (2022). What are the odds of burnt-out risk and leaving the job? Turnover intent consequences of worker burnout using a two sample New Zealand study. *International Journal of Selection and Assessment*. Advance online publication. 10.1111/ijsa.12393

[31] Hadžibajramović E, Schaufeli W, De Witte H (2020). A rasch analysis of the burnout assessment tool (BAT). PLoS ONE.

[32] Halbesleben JR, Buckley MR (2004). Burnout in organizational life. J Manag.

[33] Hancock GR, Mueller RO. Rethinking construct reliability within latent variable systems. In: Cudeck R, du Toit S, Sörbom D, editors. Structural equation modeling: Present and future-A festschrift in honor of Karl Jöreskog. Scientific Software International; 2001. pp. 195–216.

[34] Harrell FEJ. (2021). Hmisc: Harrell Miscellaneous (R package version 4.6-0) [Computer software]. https://cran.r-project.org/package=Hmisc

[35] Jodoin MG, Gierl MJ (2001). Evaluating type I error and power rates using an effect size measure with the logistic regression procedure for DIF detection. Appl Measur Educ.

[36] Kelley K. (2022). MBESS: The MBESS R Package (R package version 4.9.0) [Computer software]. https://CRAN.R-project.org/package=MBESS

[37] Kropko J, Harden JJ. (2020). coxed: Duration-Based Quantities of Interest for the Cox Proportional Hazards Model. R package version 0.3.3. https://CRAN.R-project.org/package=coxed

[38] Leiter MP (2021). Assessment of workplace social encounters: social profiles, burnout, and engagement. Int J Environ Res Public Health.

[39] Levant RF, Hall RJ, Weigold IK, McCurdy ER (2016). Construct validity evidence for the male role norms inventory-short form: a structural equation modeling approach using the bifactor model. J Couns Psychol.

[40] Linacre JM (2022). Winsteps® (Version 5.2.3) [Computer Software].

[41] Lorenzo-Seva U, Ferrando PJ. (2021). Factor (Version 12.01.02) [Computer software]. Universitat Rovira i Virgili. https://psico.fcep.urv.cat/utilitats/factor/index.html

[42] Mair P. (2018). *Modern psychometrics with R*. Springer. 10.1007/978-3-319-93177-7

[43] Mansolf M, Reise SP (2017). When and why the second-order and bifactor models are distinguishable. Intelligence.

[44] Marsh HW, Hocevar D (1988). A new, more powerful approach to multitrait-multimethod analyses: application of second-order confirmatory factor analysis. J Appl Psychol.

[45] Maslach C, Jackson SE, Leiter MP. Maslach Burnout Inventory: Manual. 4th ed. Mind Garden; 2018.

[46] Molodynski A, Lewis T, Kadhum M, Farrell SM, Lemtiri Chelieh M, De Falcão T, Masri R, Kar A, Volpe U, Moir F, Torales J, Castaldelli-Maia JM, Chau S, Wilkes C, Bhugra D (2021). Cultural variations in wellbeing, burnout and substance use amongst medical students in twelve countries. Int Rev Psychiatry.

[47] Monnapula-Mazabane P, Petersen I. Mental health stigma experiences among caregivers and service users in South Africa: a qualitative investigation. Curr Psychol. 2021;1–13. 10.1007/s12144-021-02236-y. Advance online publication. 10.1007/s12144-021-02236-y PMC8396139 34465971

[48] Oprea B, Iliescu D, De Witte H (2021). Romanian short version of the Burnout Assessment Tool: psychometric properties. Eval Health Prof.

[49] Perline R, Wright BD, Wainer H (1979). The Rasch model as additive conjoint measurement. Appl Psychol Meas.

[50] Pines AM, Ben-Ari A, Utasi A, Larson D (2002). A cross-cultural investigation of social support and burnout. Eur Psychol.

[51] R Core Team. (2021). R: A language and environment for statistical computing. R Foundation for Statistical Computing, Vienna, Austria. https://www.R-project.org/

[52] Rattrie LTB, Kittler MG, Paul KI (2020). Culture, burnout, and engagement: a meta-analysis on national cultural values as moderators in JD-R theory. Appl Psychology: Int Rev.

[53] Reise SP, Moore TM, Haviland MG (2010). Bifactor models and rotations: exploring the extent to which multidimensional data yield univocal scale scores. J Pers Assess.

[54] Rizzo M, Szekely G. (2022). energy: E-Statistics: Multivariate Inference via the Energy of Data (R package version 1.7-9) [Computer software]. https://CRAN.R-project.org/package=energy

[55] Rodriguez A, Reise SP, Haviland MG (2016). Evaluating bifactor models: calculating and interpreting statistical indices. Psychol Methods.

[56] Rodriguez A, Reise SP, Haviland MG (2016). Applying bifactor model statistical indices in the evaluation of psychological measures. J Pers Assess.

[57] Rönkkö M, Cho E (2022). An updated guideline for assessing discriminant validity. Organizational Res Methods.

[58] Sakakibara K, Shimazu A, Toyama H, Schaufeli WB (2020). Validation of the japanese version of the burnout assessment tool. Front Psychol.

[59] Schaufeli WB (2018). *Burnout in Europe: relations with national economy, governance, and culture* Research Unit Occupational & Organizational psychology and Professional Learning (internal report).

[60] Schaufeli WB. (2017). Burnout: A short socio-cultural history. In S. Neckel, A. K. Schaffner, & G. Wagner, editors, *Burnout, fatigue, exhaustion: An interdisciplinary perspective on a modern affliction* (pp. 105–127). Palgrave Macmillan. 10.1007/978-3-319-52887-8_5

[61] Schaufeli WB, de Witte H, Desart S. (2020). *Burnout Assessment Tool*. Retrieved from https://burnoutassessmenttool.be/wp-content/uploads/2020/08/Test-Manual-BAT-English-version-2.0-1.pdf 10.3390/ijerph17249495 PMC7766078 33352940

[62] Schaufeli WB, Desart S, De Witte H (2020). Burnout Assessment Tool (BAT)—Development, validity, and reliability. Int J Environ Res Public Health.

[63] Schult J, Sparfeldt JR (2016). Do non-g factors of cognitive ability tests align with specific academic achievements? A combined bifactor modeling approach. Intelligence.

[64] Schutte N, Toppinen S, Kalimo R, Schaufeli W (2000). The factorial validity of the Maslach Burnout Inventory-General Survey (MBI-GS) across occupational groups and nations. J Occup Organizational Psychol.

[65] Sinval J, Vazquez ACS, Hutz CS, Schaufeli WB, Silva S (2022). Burnout Assessment Tool (BAT): Validity evidence from Brazil and Portugal. Int J Environ Res Public Health.

[66] Sireci S. (2011). Evaluating Test and Survey Items for Bias Across Languages and Cultures. In D. Matsumoto & F. Van de Vijver, editors, *Cross-Cultural Research Methods in Psychology* (Culture and Psychology, pp. 216–240). Cambridge University Press. 10.1017/CBO9780511779381.011

[67] Smith RM (1996). A comparison of methods for determining dimensionality in Rasch measurement. Struct Equ Model.

[68] Steiner MD, Grieder SG (2020). EFAtools: an R package with fast and flexible implementations of exploratory factor analysis tools. J Open Source Softw.

[69] Storm K, Rothmann S (2003). A psychometric analysis of the Maslach Burnout Inventory-General Survey in the south african Police Service. South Afr J Psychol.

[70] Stucky BD, Edelen MO. Using hierarchical IRT models to create unidimensional measures from multidimensional data. In: Reise SP, Revicki DA, editors. Handbook of item response theory modeling: applications to typical performance assessment. Routledge; 2014. pp. 183–206.

[71] Squires A, Finlayson C, Gerchow L, Cimiotti JP, Matthews A, Schwendimann R, Griffiths P, Busse R, Heinen M, Brzostek T, Moreno-Casbas MT, Aiken LH, Sermeus W (2014). Methodological considerations when translating “burnout. Burnout Res.

[72] Vinueza-Solórzano AM, Portalanza-Chavarría CA, de Freitas CP, Schaufeli WB, De Witte H, Hutz CS, Souza Vazquez AC (2021). The ecuadorian version of the Burnout Assessment Tool (BAT): adaptation and validation. Int J Environ Res Public Health.

[73] Wiernik BM, Wilmot MP, Kostal JW (2015). How data analysis can dominate interpretations of dominant general factors. Industrial and Organizational Psychology.

[74] Wright BD, Masters GN (1990). Computation of OUTFIT and INFIT Statistics. Rasch Meas Trans.

[75] Yen WM (1984). Effects of local item dependence on the fit and equating performance of the three-parameter logistic model. Appl Psychol Meas.

[76] Zhang G, Jiang G, Hattori M, Trichtinger L. (2020). EFAutilities: Utility Functions for Exploratory Factor Analysis (R package version 2.1.1) [Computer software]. https://CRAN.R-project.org/package=EFAutilities

[77] Zumbo BD. A handbook on the theory and methods of differential item functioning (DIF): logistic regression modeling as a unitary framework for binary and likert-type (ordinal) item scores. Directorate of Human Resources Research and Evaluation, Department of National Defense; 1999.

