# Supplementary material for: Psychometric properties of the Burnout Assessment Tool across four countries
Source: BMC Public Health. 2023 May 4;23:824. doi: 10.1186/s12889-023-15604-z (PMC10161461; doi:10.1186/s12889-023-15604-z)
Supplement: Supplementary file 1 — Supplementary Material 1 [file 12889_2023_15604_MOESM1_ESM.docx]

**Online Supplement**

http://osf.io/gsaq2/?view_only=c0ed2f84c2cc4b3690b97a7fe333111c

**1. Screening Questions**

The first screening question presented cartoon pictures of four animals in a random order. These animals were a *bee*, *fox*, *panda*, and *cat*. We asked participants to click on the picture of the *bee*. The second screening question presented at the end of the biographical questionnaire asked the participants to type the word *bee*. The third screening question presented in the BAT questionnaire asked the participants to select the *rarely* response option. The final screening question asked participants to select among four options the animal presented at the start of the questionnaires. The four options were *mountains*, *fruit*, *fox*, and *bee*.

**2. Descriptive statistics**

| **Supplementary Table OS1**  *Descriptive Statistics and Reliability Coefficients for the BAT Scale Scores in the Combined and Country Sample Groups* | | | | | | | | |
| --- | --- | --- | --- | --- | --- | --- | --- | --- |
| Scale | Mean | SD | Mdn. | Skew. | Kurt. | SE | α | ω_T_ |
|  | Combined Sample | | | | | | | |
| Exhaustion | 2.96 | .74 | 2.88 | .30 | -.11 | .03 | .87 | .88 |
| Mental Distance | 2.53 | .81 | 2.60 | .20 | -.43 | .03 | .80 | .81 |
| Cognitive Impairment | 2.38 | .79 | 2.40 | .47 | .27 | .03 | .90 | .91 |
| Emotional Impairment | 1.95 | .72 | 1.90 | .79 | .59 | .03 | .83 | .83 |
| Total Burnout | 2.52 | .62 | 2.48 | .40 | .41 | .02 | .93 | .93 |
|  | Australia | | | | | | | |
| Exhaustion | 2.94 | .74 | 2.88 | .08 | -.22 | .05 | .90 | .90 |
| Mental Distance | 2.55 | .82 | 2.60 | .18 | -.45 | .06 | .84 | .85 |
| Cognitive Impairment | 2.39 | .80 | 2.20 | .55 | .33 | .06 | .93 | .93 |
| Emotional Impairment | 1.91 | .70 | 1.80 | .82 | 1.08 | .05 | .86 | .86 |
| Total Burnout | 2.51 | .64 | 2.48 | .34 | .48 | .05 | .94 | .94 |
|  | The Netherlands | | | | | | | |
| Exhaustion | 2.89 | .75 | 2.88 | .49 | .14 | .05 | .87 | .88 |
| Mental Distance | 2.39 | .81 | 2.40 | .35 | -.36 | .06 | .82 | .82 |
| Cognitive Impairment | 2.44 | .74 | 2.40 | .19 | .39 | .05 | .88 | .89 |
| Emotional Impairment | 1.94 | .69 | 1.80 | .57 | -.20 | .05 | .81 | .81 |
| Total Burnout | 2.48 | .61 | 2.43 | .62 | .73 | .04 | .93 | .93 |
|  | South Africa | | | | | | | |
| Exhaustion | 2.95 | .73 | 2.88 | .29 | -.19 | .05 | .87 | .88 |
| Mental Distance | 2.53 | .76 | 2.60 | .14 | -.14 | .05 | .74 | .74 |
| Cognitive Impairment | 2.17 | .77 | 2.00 | .62 | .05 | .05 | .90 | .90 |
| Emotional Impairment | 1.93 | .71 | 1.80 | .92 | .87 | .05 | .80 | .80 |
| Total Burnout | 2.47 | .60 | 2.43 | .39 | .31 | .04 | .92 | .92 |
|  | United States | | | | | | | |
| Exhaustion | 3.05 | .73 | 3.00 | .33 | -.26 | .05 | .87 | .87 |
| Mental Distance | 2.66 | .83 | 2.60 | .13 | -.69 | .06 | .81 | .82 |
| Cognitive Impairment | 2.51 | .80 | 2.40 | .54 | .40 | .06 | .90 | .91 |
| Emotional Impairment | 2.03 | .77 | 2.00 | .75 | .34 | .05 | .86 | .86 |
| Total Burnout | 2.63 | .60 | 2.61 | .27 | .13 | .04 | .92 | .92 |
| *Note.* SD = standard deviation, Mdn. = median, Skew. = skewness, Kurt. = kurtosis, SE = standard error of the mean, α = coefficient alpha, ω_T_ = coefficient omega total. Combined *n* = 794, Australia *n* = 200, Netherlands *n* = 199, South Africa *n* = 197, United States *n* = 198. | | | | | | | | |

**3. Pearson and distance correlation coefficients**

| **Supplementary Table OS2**  *Pearson, Distance, and Multiple Correlation Coefficients for the BAT and MBI Scale Scores for Australia* | | | | | | | | | | |
| --- | --- | --- | --- | --- | --- | --- | --- | --- | --- | --- |
|  | **Scale** | 1 | 2 | 3 | 4 | 5 | 6 | 7 | 8 | 9 |
| 1. | BAT Exhaustion | **.86** | .57 | .55 | .59 | . | .83 | .51 | .28 | .74 |
| 2. | Mental Distance | .61 | **.86** | .53 | .53 | . | .61 | .78 | .44 | .77 |
| 3. | Cognitive Impairment | .62 | .57 | **.63** | .46 | . | .52 | .48 | .35 | .55 |
| 4. | Emotional Impairment | .62 | .60 | .55 | **.64** | . | .55 | .49 | .35 | .57 |
| 5. | BAT Total | . | . | . | . | **.87** | .80 | .69 | .42 | .83 |
| 6. | MBI Exhaustion | .85 | .66 | .56 | .57 | .82 | **.87** | .60 | .24 | . |
| 7. | Cynicism | .53 | .82 | .51 | .53 | .71 | .63 | **.83** | .36 | . |
| 8. | Professional Efficacy | -.32 | -.48 | -.39 | -.40 | -.46 | -.27 | -.37 | **.51** | .33 |
| 9. | MBI Total | .77 | .82 | .59 | .61 | . | . | . | -.35 | **.89** |
| *Note.* Pearson correlation coefficients below the diagonal and distance correlation coefficients above the diagonal. Multiple correlation coefficients on the diagonal. MBI Total does not include the Professional Efficacy items. | | | | | | | | | | |
| **Supplementary Table OS3**  *Pearson, Distance, and Multiple Correlation Coefficients for the BAT and MBI Scale Scores for the Netherlands* | | | | | | | | | | |
|  | Scale | 1 | 2 | 3 | 4 | 5 | 6 | 7 | 8 | 9 |
| 1. | BAT Exhaustion | **.84** | .54 | .55 | .49 | . | .81 | .55 | .35 | .75 |
| 2. | Mental Distance | .59 | **.82** | .47 | .43 | . | .55 | .76 | .45 | .73 |
| 3. | Cognitive Impairment | .61 | .52 | **.62** | .46 | . | .50 | .44 | .45 | .52 |
| 4. | Emotional Impairment | .55 | .49 | .50 | **.56** | . | .49 | .40 | .28 | .49 |
| 5. | BAT Total | . | . | . | . | **.86** | .78 | .68 | .47 | .81 |
| 6. | MBI Exhaustion | .84 | .61 | .55 | .53 | .81 | **.85** | .60 | .36 | . |
| 7. | Cynicism | .59 | .79 | .46 | .45 | .71 | .64 | **.81** | .37 | . |
| 8. | Professional Efficacy | -.38 | -.47 | -.46 | -.32 | -.50 | -.36 | -.39 | **.54** | .40 |
| 9. | MBI Total | .79 | .77 | .56 | .54 | .84 | . | . | -.42 | **.88** |
| *Note.* Pearson correlation coefficients below the diagonal and distance correlation coefficients above the diagonal. Multiple correlation coefficients on the diagonal in bold. MBI Total does not include the Professional Efficacy items. | | | | | | | | | | |

| **Supplementary Table OS4**  *Pearson, Distance, and Multiple Correlation Coefficients for the BAT and MBI Scale Scores for South Africa* | | | | | | | | | | |
| --- | --- | --- | --- | --- | --- | --- | --- | --- | --- | --- |
|  | Scale | 1 | 2 | 3 | 4 | 5 | 6 | 7 | 8 | 9 |
| 1. | BAT Exhaustion | **.82** | .58 | .56 | .39 | . | .77 | .60 | .22 | .75 |
| 2. | Mental Distance | .65 | **.74** | .48 | .46 | . | .54 | .70 | .36 | .66 |
| 3. | Cognitive Impairment | .61 | .54 | **.69** | .47 | . | .54 | .58 | .42 | .60 |
| 4. | Emotional Impairment | .41 | .51 | .51 | **.54** | . | .43 | .44 | .32 | .47 |
| 5. | BAT Total | . | . | . | . | **.85** | .75 | .72 | .40 | .81 |
| 6. | MBI Exhaustion | .81 | .59 | .59 | .45 | .78 | **.83** | .66 | .25 | . |
| 7. | Cynicism | .64 | .73 | .62 | .48 | .76 | .70 | **.79** | .42 | . |
| 8. | Professional Efficacy | -.20 | -.38 | -.42 | -.36 | -.40 | -.23 | -.41 | **.50** | .34 |
| 9. | MBI Total | .80 | .71 | .65 | .50 | .84 | . | . | -.33 | **.85** |
| *Note.* Pearson correlation coefficients below the diagonal and distance correlation coefficients above the diagonal. Multiple correlation coefficients on the diagonal in bold. MBI Total does not include the Professional Efficacy items. | | | | | | | | | | |
|  | | | | | | | | | | |
| **Supplementary Table OS5**  *Pearson, Distance, and Multiple Correlation Coefficients for the BAT and MBI Scale Scores for the United States* | | | | | | | | | | |
|  | Scale | 1 | 2 | 3 | 4 | 5 | 6 | 7 | 8 | 9 |
| 1. | BAT Exhaustion | **.81** | .44 | .39 | . | .79 | .78 | .52 | .21 | .70 |
| 2. | Mental Distance | .47 | **.81** | .45 | . | .72 | .58 | .77 | .37 | .72 |
| 3. | Cognitive Impairment | .45 | .48 | **.50** | . | .68 | .39 | .43 | .26 | .45 |
| 4. | Emotional Impairment | .51 | .34 | .43 | **.51** | .67 | .46 | .38 | .21 | .46 |
| 5. | BAT Total | . | . | . | . | **.83** | .77 | .70 | .34 | .81 |
| 6. | MBI Exhaustion | .81 | .60 | .43 | .50 | .79 | **.85** | .68 | .19 | . |
| 7. | Cynicism | .55 | .80 | .47 | .41 | .73 | .71 | **.83** | .37 | . |
| 8. | Professional Efficacy | -.18 | -.35 | -.23 | -.17 | -.30 | -.11 | -.33 | **.36** | .29 |
| 9. | MBI Total | .74 | .76 | .49 | .49 | .82 | . | . | -.24 | **.87** |
| *Note.* Pearson correlation coefficients below the diagonal and distance correlation coefficients above the diagonal. Multiple correlation coefficients on the diagonal in bold. MBI Total does not include Professional Efficacy items. | | | | | | | | | | |

**4. BAT bifactor models**

| **Supplementary Table OS6**  *Target Rotated Bifactor Model and Item Explained Common Variance for the Combined Sample* | | | | | | |
| --- | --- | --- | --- | --- | --- | --- |
| Item | General | Exhaustion | Mental | Cognitive | Emotional | I-ECV |
| EX1 | **.65** | **.47** | .05 | .01 | -.03 | **.65** |
| EX2 | .06 | **.69** | .10 | .05 | **.12** | .01 |
| EX3 | **.67** | **.43** | -.04 | .00 | -.02 | **.71** |
| EX4 | **.54** | **.40** | .01 | **-.19** | **.10** | **.59** |
| EX5 | **.75** | **.17** | -.03 | .00 | **-.11** | **.93** |
| EX6 | **.72** | **.24** | .02 | **.14** | -.01 | **.87** |
| EX7 | **.69** | **.38** | -.01 | .06 | .09 | **.75** |
| EX8 | **.70** | **.48** | -.06 | -.06 | -.04 | **.67** |
| MD1 | **.85** | -.05 | **.26** | -.07 | **-.17** | **.88** |
| MD2 | **.57** | **-.19** | .13 | -.06 | -.02 | **.85** |
| MD3 | **.48** | **.16** | **.64** | -.09 | **.12** | .34 |
| MD4 | **.60** | **-.13** | **.53** | -.02 | -.04 | **.55** |
| MD5 | **.48** | .11 | **.49** | **.13** | .10 | .45 |
| CI1 | **.67** | -.05 | .01 | **.53** | **-.08** | **.61** |
| CI2 | **.68** | **.10** | .02 | **.54** | .06 | **.60** |
| CI3 | **.67** | -.03 | .00 | **.60** | .02 | **.55** |
| CI4 | **.69** | -.03 | -.06 | **.64** | -.01 | **.53** |
| CI5 | **.55** | -.02 | -.02 | **.41** | **.21** | **.59** |
| EI1 | **.55** | .01 | -.04 | .04 | **.61** | .44 |
| EI2 | **.52** | -.01 | .06 | -.01 | **.60** | .43 |
| EI3 | **.46** | .09 | .06 | .08 | **.47** | .47 |
| EI4 | **.66** | .09 | .04 | .03 | **.43** | **.69** |
| EI5 | **.45** | .01 | .01 | .02 | **.70** | .29 |
| *Note*. EX = BAT Exhaustion, MD = Mental Distance, CI = Cognitive Impairment, EI = Emotional Impairment, General = General Factor, Exhaustion = BAT Exhaustion, Mental = Mental Distance, Cognitive = Cognitive Impairment, Emotional = Emotional Impairment, I-ECV = item explained common variance. Statistically significant factor loadings and item explained common variance > .50 in bold. | | | | | | |

| **Supplementary Table OS7**  *Target Rotated Bifactor Model and Item Explained Common Variance for Australia* | | | | | | |
| --- | --- | --- | --- | --- | --- | --- |
| Item | General | Exhaustion | Mental | Cognitive | Emotional | I-ECV |
| EX1 | **.56** | **.58** | .10 | .07 | .02 | .47 |
| EX2 | **.31** | **.56** | -.06 | -.02 | .04 | .23 |
| EX3 | **.65** | **.39** | .13 | -.03 | .11 | **.70** |
| EX4 | **.52** | **.44** | -.04 | -.20 | .10 | **.52** |
| EX5 | **.72** | .21 | -.03 | .09 | -.12 | **.88** |
| EX6 | **.73** | .24 | -.08 | .13 | -.02 | **.87** |
| EX7 | **.73** | .30 | -.14 | .11 | .00 | **.81** |
| EX8 | **.64** | **.49** | -.01 | .01 | .03 | **.63** |
| MD1 | **.85** | -.06 | .27 | -.13 | -.21 | **.84** |
| MD2 | **.58** | -.22 | .06 | -.16 | .03 | **.81** |
| MD3 | **.65** | .10 | **.43** | -.12 | .09 | **.66** |
| MD4 | **.58** | -.14 | .49 | -.03 | -.06 | **.57** |
| MD5 | **.50** | .06 | **.58** | .23 | .15 | .37 |
| CI1 | **.68** | .01 | -.05 | **.48** | -.13 | **.65** |
| CI2 | **.67** | .06 | .05 | **.59** | .07 | **.56** |
| CI3 | **.63** | -.04 | .05 | **.59** | .00 | **.53** |
| CI4 | **.67** | .00 | -.04 | **.64** | -.05 | **.52** |
| CI5 | **.64** | .00 | .02 | **.43** | .11 | **.68** |
| EI1 | **.59** | .09 | -.08 | -.06 | **.48** | **.58** |
| EI2 | **.56** | .00 | .09 | -.03 | **.54** | **.52** |
| EI3 | **.58** | .00 | .08 | .10 | **.30** | **.76** |
| EI4 | **.58** | .14 | .07 | .09 | **.42** | **.62** |
| EI5 | **.58** | .01 | -.04 | -.05 | **.61** | .48 |
| *Note.* EX = BAT Exhaustion, MD = Mental Distance, CI = Cognitive Impairment, EI = Emotional Impairment, General = General Factor, Exhaustion = BAT Exhaustion, Mental = Mental Distance, Cognitive = Cognitive Impairment, Emotional = Emotional Impairment, I-ECV = item explained common variance. Statistically significant factor loadings and item explained common variance > .50 in bold. | | | | | | |

| **Supplementary Table OS8**  *Target Rotated Bifactor Model and Item Explained Common Variance for the Netherlands* | | | | | | |
| --- | --- | --- | --- | --- | --- | --- |
| Item | General | Exhaustion | Mental | Cognitive | Emotional | I-ECV |
| EX1 | **.64** | **.38** | .06 | .01 | -.02 | **.73** |
| EX2 | .36 | .43 | -.23 | -.08 | .06 | .34 |
| EX3 | **.68** | .36 | -.02 | -.07 | -.05 | **.77** |
| EX4 | **.71** | -.19 | -.13 | **-.27** | .02 | **.80** |
| EX5 | **.67** | .17 | .15 | .10 | -.13 | **.85** |
| EX6 | **.61** | .43 | .07 | .18 | .04 | **.62** |
| EX7 | **.73** | .24 | -.05 | .02 | .08 | **.89** |
| EX8 | **.70** | .35 | -.02 | -.04 | -.11 | **.78** |
| MD1 | **.68** | .17 | **.44** | .03 | -.11 | **.66** |
| MD2 | **.47** | **-.38** | .29 | -.09 | -.03 | .47 |
| MD3 | **.54** | .10 | **.54** | -.05 | .05 | .48 |
| MD4 | **.54** | -.13 | **.52** | .10 | .05 | .49 |
| MD5 | **.50** | .06 | **.41** | .07 | .11 | **.57** |
| CI1 | **.55** | .01 | .07 | **.55** | -.04 | .49 |
| CI2 | **.63** | .15 | -.06 | **.41** | .04 | **.67** |
| CI3 | **.62** | .03 | .01 | **.58** | .02 | **.53** |
| CI4 | **.59** | .08 | .03 | **.67** | -.02 | .43 |
| CI5 | **.53** | -.17 | -.01 | **.33** | .13 | **.64** |
| EI1 | **.49** | .05 | -.01 | .02 | **.58** | .42 |
| EI2 | **.54** | -.18 | -.06 | .01 | .36 | **.63** |
| EI3 | **.49** | .00 | -.06 | .02 | **.35** | **.65** |
| EI4 | **.55** | .15 | .21 | .10 | **.44** | **.53** |
| EI5 | **.40** | -.09 | -.01 | -.04 | **.69** | .25 |
| *Note.* EX = BAT Exhaustion, MD = Mental Distance, CI = Cognitive Impairment, EI = Emotional Impairment, General = General Factor, Exhaustion = BAT Exhaustion, Mental = Mental Distance, Cognitive = Cognitive Impairment, Emotional = Emotional Impairment, I-ECV = item explained common variance. Statistically significant factor loadings and item explained common variance > .50 in bold. | | | | | | |

| **Supplementary Table OS9**  *Target Rotated Bifactor Model and Item Explained Common Variance for South Africa* | | | | | | |
| --- | --- | --- | --- | --- | --- | --- |
| Item | General | Exhaustion | Mental | Cognitive | Emotional | I-ECV |
| EX1 | **.68** | **.34** | -.04 | -.06 | .02 | .79 |
| EX2 | -.04 | **.55** | .12 | .05 | .00 | .00 |
| EX3 | **.64** | **.41** | -.06 | .07 | -.13 | .68 |
| EX4 | **.48** | **.59** | .08 | -.05 | .05 | .39 |
| EX5 | **.69** | .23 | -.02 | .03 | -.12 | .87 |
| EX6 | **.73** | .24 | .08 | .17 | -.07 | .85 |
| EX7 | **.65** | **.38** | .09 | .09 | -.01 | .72 |
| EX8 | **.66** | **.41** | -.05 | -.07 | .02 | .71 |
| MD1 | **.87** | -.11 | -.03 | -.11 | -.12 | .95 |
| MD2 | **.52** | -.02 | .06 | -.03 | -.02 | .98 |
| MD3 | **.43** | .04 | .60 | -.11 | .07 | .33 |
| MD4 | **.60** | -.06 | .31 | -.14 | .07 | .75 |
| MD5 | **.41** | .25 | .40 | .08 | .12 | .41 |
| CI1 | **.67** | .03 | .07 | **.47** | -.06 | .67 |
| CI2 | **.67** | .09 | .02 | **.52** | .03 | .62 |
| CI3 | **.64** | .04 | -.04 | **.49** | .06 | .62 |
| CI4 | **.66** | .02 | -.05 | **.61** | .06 | .54 |
| CI5 | **.49** | -.10 | -.20 | **.34** | .22 | .54 |
| EI1 | **.52** | -.17 | -.07 | .06 | **.57** | .43 |
| EI2 | **.46** | -.08 | -.05 | .03 | **.53** | .42 |
| EI3 | **.36** | -.01 | .08 | .05 | **.47** | .36 |
| EI4 | **.57** | .12 | .07 | -.05 | **.40** | .65 |
| EI5 | .27 | .07 | .19 | .12 | **.65** | .13 |
| *Note.* EX = BAT Exhaustion, MD = Mental Distance, CI = Cognitive Impairment, EI = Emotional Impairment, General = General Factor, Exhaustion = BAT Exhaustion, Mental = Mental Distance, Cognitive = Cognitive Impairment, Emotional = Emotional Impairment, I-ECV = item explained common variance. Statistically significant factor loadings and item explained common variance > .50 in bold. | | | | | | |

| **Supplementary Table OS10**  *Target Rotated Bifactor Model and Item Explained Common Variance for the United States* | | | | | | |
| --- | --- | --- | --- | --- | --- | --- |
| Item | General | Exhaustion | Mental | Cognitive | Emotional | I-ECV |
| EX1 | **.52** | **.59** | .09 | .07 | -.02 | .42 |
| EX2 | .02 | **.54** | -.06 | .04 | .16 | .00 |
| EX3 | **.53** | **.61** | .01 | .05 | .03 | .43 |
| EX4 | **.52** | **.45** | .01 | -.16 | .05 | **.55** |
| EX5 | **.70** | .18 | .03 | -.10 | -.04 | **.92** |
| EX6 | **.72** | .06 | -.02 | .02 | .02 | **.99** |
| EX7 | **.63** | **.37** | -.06 | -.06 | .13 | **.71** |
| EX8 | **.59** | **.58** | -.03 | -.03 | -.04 | **.51** |
| MD1 | **.77** | .01 | **.45** | -.04 | **-.20** | **.71** |
| MD2 | **.56** | -.22 | .14 | .07 | -.11 | **.79** |
| MD3 | **.38** | .13 | **.57** | -.07 | .13 | .29 |
| MD4 | **.46** | -.11 | **.63** | .04 | -.10 | .34 |
| MD5 | **.38** | .04 | **.56** | .17 | .08 | .29 |
| CI1 | **.54** | -.07 | .11 | **.66** | -.05 | .39 |
| CI2 | **.51** | .16 | .15 | **.65** | .12 | .35 |
| CI3 | **.59** | -.08 | -.03 | **.62** | -.01 | .47 |
| CI4 | **.63** | -.08 | -.08 | **.63** | -.04 | .49 |
| CI5 | **.47** | .01 | -.09 | **.39** | .21 | **.52** |
| EI1 | **.43** | .00 | -.04 | .09 | **.67** | .29 |
| EI2 | **.43** | -.01 | .02 | -.06 | **.60** | .34 |
| EI3 | **.43** | .19 | .05 | .08 | **.50** | .39 |
| EI4 | **.58** | .15 | .00 | .02 | **.49** | **.56** |
| EI5 | **.42** | -.05 | -.09 | .03 | **.66** | .29 |
| *Note.* EX = BAT Exhaustion, MD = Mental Distance, CI = Cognitive Impairment, EI = Emotional Impairment, General = General Factor, Exhaustion = BAT Exhaustion, Mental = Mental Distance, Cognitive = Cognitive Impairment, Emotional = Emotional Impairment, I-ECV = item explained common variance. Statistically significant factor loadings and item explained common variance > .50 in bold. | | | | | | |

**5. Proportional reduction in mean squared errors and external incremental validity**

| **Supplementary Table OS11**  *Proportional Reduction in Mean Squared Error for the Combined Sample* | | | | |
| --- | --- | --- | --- | --- |
| Scale | Primary | CI | General | Primary and General |
| Exhaustion | .924 | [.913, .933] | .588 | .961 |
| Mental Distance | .893 | [.877, .907] | .487 | .944 |
| Cognitive Impairment | .957 | [.948, .968] | .540 | .978 |
| Emotional Impairment | .900 | [.881, .911] | .397 | .947 |
| *Note*. Primary = group factors, CI = 95% confidence interval, General = unidimensional model, Primary and General = group factors and unidimensional model. | | | | |

| **Supplementary Table OS12**  *Proportional Reduction in Mean Squared Error for Australia* | | | | |
| --- | --- | --- | --- | --- |
| Scale | Primary | CI | General | Primary and General |
| Exhaustion | .919 | [.896, .933] | .728 | .958 |
| Mental Distance | .896 | [.860, .919] | .606 | .945 |
| Cognitive Impairment | .949 | [.933, .961] | .664 | .974 |
| Emotional Impairment | .898 | [.860, .919] | .609 | .946 |
| *Note*. Primary = group factors, CI = 95% confidence interval, General = unidimensional model, Primary and General = group factors and unidimensional model. | | | | |

| **Supplementary Table OS13**  *Proportional Reduction in Mean Squared Error for the Netherlands* | | | | |
| --- | --- | --- | --- | --- |
| Scale | Primary | CI | General | Primary and General |
| Exhaustion | .908 | [.877, .926] | .722 | .952 |
| Mental Distance | .860 | [.811, .886] | .550 | .925 |
| Cognitive Impairment | .922 | [.891, .942] | .641 | .959 |
| Emotional Impairment | .856 | [.791 .885] | .473 | .922 |
| *Note*. Primary = group factors, CI = 95% confidence interval, General = unidimensional model, Primary and General = group factors and unidimensional model. | | | | |

| **Supplementary Table OS14**  *Proportional Reduction in Mean Squared Error for South Africa* | | | | |
| --- | --- | --- | --- | --- |
| Scale | Primary | CI | General | Primary and General |
| Exhaustion | .913 | [.886, .929] | .728 | .955 |
| Mental Distance | .905 | [.880, .991] | .725 | .950 |
| Cognitive Impairment | .925 | [.900, .949] | .676 | .961 |
| Emotional Impairment | .834 | [.772 .864] | .380 | .910 |
| *Note*. Primary = group factors, CI = 95% confidence interval, General = unidimensional model, Primary and General = group factors and unidimensional model. | | | | |

| **Supplementary Table OS15**  *Proportional Reduction in Mean Squared Error for the United States* | | | | |
| --- | --- | --- | --- | --- |
| Scale | Primary | CI | General | Primary and General |
| Exhaustion | .912 | [.890, .925] | .633 | .954 |
| Mental Distance | .888 | [.854, .907] | .446 | .940 |
| Cognitive Impairment | .930 | [.909, .944] | .564 | .964 |
| Emotional Impairment | .886 | [.847 .905] | .483 | .940 |
| *Note*. Primary = group factors, CI = 95% confidence interval, General = unidimensional model, Primary and General = group factors and unidimensional model. | | | | |

| **Supplementary Table OS16**  *External Incremental Variance for the Combined Sample* | | |
| --- | --- | --- |
| External Variable | Difference | CI |
| Satisfaction | .50 | [.41, .61] |
| Complexity | .40 | [.23, .57] |
| Turnover | -.01 | [-.07, .06] |
| *Note*. Difference = difference in correlation from primary factors and unidimensional model general factor, CI = 95% confidence intervals. | | |

| **Supplementary Table OS17**  *External Incremental Variance for Australia* | | |
| --- | --- | --- |
| External Variable | Difference | CI |
| Satisfaction | .45 | [.31, .67] |
| Complexity | .39 | [.14, .79] |
| Turnover | -.02 | [-.14, .14] |
| *Note*. Difference = difference in correlation from primary factors and unidimensional model general factor, CI = 95% confidence intervals. | | |

| **Supplementary Table OS18**  *External Incremental Variance for the Netherlands* | | |
| --- | --- | --- |
| External Variable | Difference | CI |
| Satisfaction | .34 | [.14, .71] |
| Complexity | .45 | [.18, .85] |
| Turnover | .00 | [-.12, .16] |
| *Note*. Difference = difference in correlation from primary factors and unidimensional model general factor, CI = 95% confidence intervals. | | |

| **Supplementary Table OS19**  *External Incremental Variance for South Africa* | | |
| --- | --- | --- |
| External Variable | Difference | CI |
| Satisfaction | .43 | [.27, .69] |
| Complexity | .32 | [.07, .80] |
| Turnover | -.03 | [-.14, .14] |
| *Note*. Difference = difference in correlation from primary factors and unidimensional model general factor, CI = 95% confidence intervals. | | |

| **Supplementary Table OS20**  *External Incremental Variance for the United States* | | |
| --- | --- | --- |
| External Variable | Difference | CI |
| Satisfaction | .71 | [.52, .92] |
| Complexity | .04 | [-.08, .18] |
| Turnover | .68 | [.38, 1.03] |
| *Note*. Difference = difference in correlation from primary factors and unidimensional model general factor, CI = 95% confidence intervals. | | |

**6. BAT and MBI bifactor models**

| **Supplementary Table OS21**  *Target Rotated Bifactor Model and Item Explained Common Variance for the BAT and MBI Items for the Combined Sample* | | | | | | | | | | | | | | | |
| --- | --- | --- | --- | --- | --- | --- | --- | --- | --- | --- | --- | --- | --- | --- | --- |
| Item | G | BEX | | MD | | CI | | EI | | MEX | | CY | | PE | I-ECV |
| BEX1 | **.63** | **.34** | | .01 | | .04 | | .00 | | **.25** | | .07 | | -.01 | **.68** |
| BEX2 | .16 | **.57** | | .20 | | -.05 | | .04 | | .08 | | .01 | | -.11 | .06 |
| BEX3 | **.65** | .29 | | -.02 | | .04 | | -.01 | | **.24** | | -.07 | | .00 | **.74** |
| BEX4 | **.54** | .37 | | -.06 | | -.14 | | .10 | | .05 | | -.16 | | -.10 | **.59** |
| BEX5 | **.71** | .12 | | -.22 | | .07 | | -.09 | | .13 | | -.13 | | .06 | **.82** |
| BEX6 | **.68** | .22 | | -.05 | | **.18** | | .01 | | -.03 | | -.06 | | .05 | **.84** |
| BEX7 | **.67** | .35 | | -.05 | | .09 | | .06 | | .00 | | -.18 | | -.02 | **.73** |
| BEX8 | **.66** | .33 | | -.04 | | .02 | | -.02 | | **.32** | | -.09 | | -.07 | **.66** |
| MD1 | **.80** | -.03 | | -.28 | | .01 | | -.10 | | -.08 | | .20 | | .05 | **.82** |
| MD2 | **.51** | -.13 | | -.17 | | .01 | | .03 | | -.14 | | .03 | | .01 | **.79** |
| MD3 | **.60** | .10 | | .07 | | **-.19** | | .06 | | -.19 | | .22 | | -.05 | **.71** |
| MD4 | **.63** | -.12 | | -.03 | | -.05 | | -.03 | | -.21 | | .21 | | .01 | **.78** |
| MD5 | **.62** | -.04 | | .36 | | -.03 | | .00 | | -.17 | | .15 | | .03 | **.66** |
| CI1 | **.58** | -.03 | | -.05 | | **.58** | | -.04 | | -.02 | | .04 | | **.08** | .48 |
| CI2 | **.61** | .09 | | .05 | | **.56** | | .07 | | .02 | | .02 | | .07 | **.52** |
| CI3 | **.58** | -.01 | | -.01 | | **.61** | | .04 | | -.06 | | -.03 | | .07 | .47 |
| CI4 | **.59** | -.02 | | -.02 | | **.68** | | .01 | | .01 | | -.06 | | .06 | .43 |
| CI5 | **.46** | .04 | | -.01 | | **.43** | | **.22** | | -.04 | | -.07 | | .10 | .46 |
| EI1 | **.45** | .03 | | -.04 | | .09 | | **.62** | | .04 | | .01 | | .05 | .34 |
| EI2 | **.44** | .06 | | -.02 | | .03 | | **.56** | | -.02 | | .02 | | .10 | .37 |
| EI3 | **.45** | .04 | | .03 | | .07 | | **.43** | | -.01 | | -.01 | | -.05 | **.50** |
| EI4 | **.60** | .03 | | .04 | | .04 | | **.40** | | .04 | | -.06 | | .05 | **.68** |
| EI5 | **.40** | -.03 | | .05 | | .02 | | **.66** | | -.01 | | -.10 | | .00 | .26 |
| MEX1 | **.74** | **.19** | | .03 | | -.03 | | .08 | | **.46** | | .07 | | -.08 | **.67** |
| MEX2 | **.71** | .20 | | .01 | | -.04 | | .04 | | **.44** | | -.04 | | **-.12** | **.67** |
| MEX3 | **.79** | .10 | | -.08 | | .00 | | -.06 | | .30 | | -.10 | | -.04 | **.84** |
| MEX4 | **.75** | .17 | | -.05 | | .03 | | -.05 | | **.29** | | -.04 | | -.08 | **.82** |
| MEX5 | **.75** | **.22** | | .01 | | -.06 | | .01 | | **.35** | | **.10** | | **-.10** | **.74** |
| CY1 | **.75** | -.14 | | -.15 | | -.04 | | -.08 | | .04 | | .46 | | .01 | **.68** |
| CY2 | **.78** | -.12 | | -.16 | | -.04 | | -.10 | | .04 | | **.46** | | .01 | **.70** |
| CY3 | **.43** | .00 | | .05 | | -.05 | | -.01 | | -.09 | | .10 | | **-.26** | **.68** |
| CY4 | **.71** | -.11 | | **.46** | | -.09 | | .01 | | -.09 | | .19 | | .01 | **.65** |
| CY5 | **.68** | -.19 | | **.31** | | .02 | | .03 | | -.01 | | .18 | | .19 | **.69** |
| PE1 | .11 | .15 | | .02 | | .07 | | **.17** | | -.09 | | .08 | | **.52** | .03 |
| PE2 | **.40** | -.14 | | .10 | | -.03 | | -.02 | | -.10 | | .02 | | **.58** | .30 |
| PE3 | **.18** | .13 | | .02 | | **.18** | | .09 | | .03 | | -.02 | | **.67** | .06 |
| PE4 | **.37** | **-.31** | | -.10 | | -.07 | | -.03 | | .01 | | .05 | | **.45** | .31 |
| PE5 | **.44** | -.31 | | -.02 | | -.09 | | -.10 | | -.06 | | -.08 | | **.57** | .30 |
| PE6 | **.25** | .16 | | -.05 | | **.22** | | .02 | | -.07 | | .00 | | **.69** | .10 |
| *Note*. BEX = BAT Exhaustion, MD = Mental Distance, CI = Cognitive Impairment, EI = Emotional Impairment, MEX = MBI Exhaustion, CY = Cynicism, PE = Professional Efficacy, G = General Factor, I-ECV = item-explained common variance. Shown factor loadings are statistically significant at *p* < .001. Statistically significant factor loadings and item explained common variance > .50 in bold. | | | | | | | | | | | | | | | |
| **Supplementary Table OS22**  *Target Rotated Bifactor Model and Item Explained Common Variance for the BAT and MBI Items for Australia* | | | | | | | | | | | | | | | |
|  | G | | BEX | | MD | | CI | | EI | | MEX | | CY | PE | I-ECV |
| BEX1 | **.63** | | .36 | | -.06 | | .03 | | -.01 | | .27 | | .04 | -.07 | **.66** |
| BEX2 | **.31** | | .54 | | .04 | | -.02 | | .04 | | .23 | | -.02 | .07 | .22 |
| BEX3 | **.73** | | .20 | | -.09 | | -.08 | | .09 | | .16 | | -.04 | -.02 | **.86** |
| BEX4 | **.51** | | .41 | | .03 | | -.16 | | .15 | | .14 | | -.10 | -.05 | **.52** |
| BEX5 | **.72** | | .17 | | .02 | | .15 | | -.08 | | .01 | | -.21 | -.05 | **.83** |
| BEX6 | **.69** | | .36 | | .03 | | .20 | | .04 | | -.09 | | -.07 | -.01 | **.72** |
| BEX7 | **.70** | | .36 | | -.04 | | .16 | | .05 | | -.02 | | -.17 | -.01 | **.72** |
| BEX8 | **.72** | | .28 | | -.17 | | -.03 | | .00 | | .27 | | -.16 | -.05 | **.71** |
| MD1 | **.84** | | -.05 | | **.37** | | -.02 | | -.11 | | -.09 | | .02 | -.02 | **.81** |
| MD2 | **.51** | | -.12 | | .19 | | -.01 | | .14 | | -.07 | | -.01 | -.05 | **.76** |
| MD3 | **.70** | | .07 | | .24 | | -.11 | | .09 | | .06 | | **.24** | .13 | **.75** |
| MD4 | **.60** | | -.14 | | .33 | | .00 | | -.02 | | -.09 | | .24 | .10 | **.64** |
| MD5 | **.65** | | -.13 | | -.17 | | .06 | | .01 | | -.07 | | **.42** | .16 | **.63** |
| CI1 | **.62** | | .09 | | .02 | | **.55** | | -.08 | | -.13 | | -.07 | .01 | **.53** |
| CI2 | **.66** | | .03 | | -.06 | | **.60** | | .05 | | .01 | | .05 | .07 | **.54** |
| CI3 | **.59** | | -.05 | | .01 | | **.64** | | .04 | | .02 | | .05 | .03 | .46 |
| CI4 | **.62** | | .01 | | -.06 | | **.67** | | -.02 | | -.01 | | -.06 | .09 | .45 |
| CI5 | **.57** | | .05 | | .02 | | **.50** | | **.17** | | .03 | | -.02 | .11 | **.52** |
| EI1 | **.54** | | .11 | | .01 | | .00 | | **.51** | | .00 | | -.07 | .00 | **.51** |
| EI2 | **.54** | | .09 | | -.01 | | .00 | | **.52** | | -.08 | | .05 | .17 | .48 |
| EI3 | **.58** | | -.07 | | .08 | | .12 | | **.33** | | -.01 | | .04 | .01 | **.71** |
| EI4 | **.60** | | .15 | | -.09 | | .07 | | **.40** | | -.05 | | .10 | .04 | **.63** |
| EI5 | **.53** | | -.04 | | .02 | | .02 | | **.71** | | .07 | | -.08 | .01 | .35 |
| MEX1 | **.76** | | **.17** | | -.06 | | -.03 | | .05 | | **.49** | | .03 | -.08 | **.67** |
| MEX2 | **.74** | | .16 | | -.07 | | -.12 | | .04 | | **.46** | | -.06 | -.04 | **.68** |
| MEX3 | **.82** | | .10 | | -.02 | | .03 | | -.10 | | .23 | | -.13 | -.07 | **.87** |
| MEX4 | **.73** | | .28 | | .00 | | .03 | | -.09 | | .31 | | -.06 | -.08 | **.74** |
| MEX5 | **.78** | | **.19** | | .04 | | .01 | | -.01 | | **.39** | | .06 | -.11 | **.75** |
| CY1 | **.73** | | -.19 | | **.35** | | -.06 | | -.07 | | -.08 | | .25 | -.03 | **.70** |
| CY2 | **.81** | | -.19 | | **.32** | | -.10 | | **-.16** | | -.01 | | .25 | -.06 | **.73** |
| CY3 | **.45** | | -.10 | | .23 | | -.07 | | -.09 | | .08 | | .14 | -.22 | **.58** |
| CY4 | **.69** | | -.08 | | -.11 | | -.04 | | .03 | | -.01 | | **.65** | .05 | **.51** |
| CY5 | **.66** | | -.09 | | -.10 | | .08 | | .05 | | -.03 | | **.57** | .06 | **.55** |
| PE1 | .06 | | .13 | | .22 | | .17 | | **.19** | | .09 | | .07 | **.60** | .01 |
| PE2 | **.41** | | -.03 | | -.13 | | -.02 | | .04 | | -.21 | | .16 | **.52** | .31 |
| PE3 | .22 | | .07 | | .04 | | .11 | | .03 | | .12 | | -.13 | **.70** | .08 |
| PE4 | **.50** | | -.32 | | -.11 | | -.17 | | -.06 | | -.11 | | -.05 | **.44** | .42 |
| PE5 | **.49** | | -.21 | | -.17 | | -.17 | | -.03 | | -.23 | | .07 | **.49** | .38 |
| PE6 | .22 | | .14 | | .09 | | **.25** | | .00 | | -.01 | | -.05 | **.67** | .09 |
| *Note*. BEX = BAT Exhaustion, MD = Mental Distance, CI = Cognitive Impairment, EI = Emotional Impairment, MEX = MBI Exhaustion, CY = Cynicism, PE = Professional Efficacy, G = General Factor, I-ECV = item-explained common variance. Shown factor loadings are statistically significant at *p* < .001. Statistically significant factor loadings and item explained common variance > .50 in bold. Professional Efficacy items are reverse scored. | | | | | | | | | | | | | | | |

| **Supplementary Table OS23**  *Target Rotated Bifactor Model and Item Explained Common Variance for the BAT and MBI Items for the Netherlands* | | | | | | | | | |
| --- | --- | --- | --- | --- | --- | --- | --- | --- | --- |
|  | G | BEX | MD | CI | EI | MEX | CY | PE | I-ECV |
| BEX1 | **.68** | .23 | -.18 | -.01 | .01 | .10 | .03 | .05 | **.82** |
| BEX2 | **.31** | **.46** | -.25 | -.02 | .07 | .04 | -.03 | -.04 | .26 |
| BEX3 | **.65** | .31 | -.08 | .01 | -.01 | **.26** | .00 | -.10 | **.69** |
| BEX4 | **.48** | .30 | .37 | -.05 | .23 | .15 | -.06 | .03 | .42 |
| BEX5 | **.75** | .02 | .03 | .06 | -.12 | .20 | -.23 | .06 | **.83** |
| BEX6 | **.69** | .28 | -.20 | .13 | -.02 | -.14 | -.05 | .02 | **.76** |
| BEX7 | **.66** | **.38** | .03 | .11 | .13 | .02 | -.06 | -.04 | **.70** |
| BEX8 | **.68** | .33 | -.09 | .03 | -.08 | **.35** | .01 | -.03 | **.65** |
| MD1 | **.83** | -.06 | .12 | -.07 | -.14 | -.11 | -.08 | -.02 | **.92** |
| MD2 | **.40** | -.13 | .54 | -.04 | .08 | .01 | .03 | .08 | .33 |
| MD3 | **.65** | -.11 | .16 | -.08 | .02 | -.07 | .09 | -.03 | **.88** |
| MD4 | **.62** | -.12 | .34 | .08 | .02 | -.15 | .19 | -.01 | **.66** |
| MD5 | **.59** | -.07 | .07 | .03 | .06 | .01 | **.49** | .15 | **.56** |
| CI1 | **.55** | -.04 | -.02 | **.52** | -.02 | -.05 | -.02 | .14 | **.51** |
| CI2 | **.59** | .20 | -.08 | **.44** | .07 | -.10 | -.04 | .08 | **.57** |
| CI3 | **.60** | .03 | -.01 | **.60** | .04 | -.04 | -.04 | .07 | .49 |
| CI4 | **.61** | -.06 | -.10 | **.63** | -.02 | -.01 | -.08 | .03 | .47 |
| CI5 | **.39** | .05 | .19 | **.50** | .20 | .16 | .05 | .08 | .30 |
| EI1 | **.46** | .11 | -.09 | .02 | **.61** | -.03 | .01 | .13 | .34 |
| EI2 | **.40** | .17 | .20 | .14 | **.45** | .01 | .02 | .01 | .36 |
| EI3 | **.43** | .07 | .06 | .10 | **.40** | .04 | -.02 | -.04 | **.51** |
| EI4 | **.61** | .00 | -.07 | .07 | **.39** | -.03 | .06 | -.03 | **.69** |
| EI5 | **.36** | -.07 | .01 | -.02 | **.73** | .05 | -.01 | -.03 | .19 |
| MEX1 | **.76** | .16 | -.08 | -.07 | .11 | **.40** | -.05 | -.02 | **.73** |
| MEX2 | **.68** | .30 | -.03 | .00 | .04 | **.45** | .06 | -.11 | **.60** |
| MEX3 | **.77** | .02 | -.02 | .03 | -.06 | .36 | -.12 | -.02 | **.80** |
| MEX4 | **.73** | .10 | .03 | .10 | -.08 | **.35** | -.03 | -.01 | **.78** |
| MEX5 | **.80** | .08 | -.09 | -.12 | .03 | **.32** | -.01 | -.04 | **.82** |
| CY1 | **.78** | -.14 | .13 | -.19 | -.17 | -.18 | .13 | -.13 | **.78** |
| CY2 | **.80** | -.09 | .16 | -.16 | **-.23** | -.20 | .16 | -.14 | **.77** |
| CY3 | **.42** | -.16 | .23 | -.04 | .05 | -.01 | .15 | -.16 | **.57** |
| CY4 | **.70** | -.02 | .07 | -.07 | .05 | .04 | **.55** | .08 | **.61** |
| CY5 | **.66** | .00 | .01 | .02 | .08 | -.02 | **.59** | .12 | **.54** |
| PE1 | **.37** | .17 | -.02 | -.08 | .03 | -.15 | -.16 | **.53** | .28 |
| PE2 | **.43** | -.12 | .08 | -.04 | -.04 | .08 | .17 | **.64** | .28 |
| PE3 | .17 | .21 | -.01 | .21 | .05 | .01 | .01 | **.62** | .06 |
| PE4 | **.32** | -.38 | -.04 | .01 | .08 | -.04 | .05 | **.32** | .29 |
| PE5 | **.41** | -.32 | .10 | .03 | .00 | .14 | .14 | **.57** | .26 |
| PE6 | **.40** | .20 | -.06 | .23 | -.02 | -.23 | -.14 | **.56** | .25 |
| *Note.* BEX = BAT Exhaustion, MD = Mental Distance, CI = Cognitive Impairment, EI = Emotional Impairment, MEX = MBI Exhaustion, CY = Cynicism, PE = Professional Efficacy, G = General Factor, I-ECV = item-explained common variance. Shown factor loadings are statistically significant at *p* < .001. Statistically significant factor loadings and item explained common variance > .50 in bold. Professional Efficacy items are reverse scored. | | | | | | | | | |

| **Supplementary Table OS24**  *Target Rotated Bifactor Model and Item Explained Common Variance for the BAT and MBI Items for South Africa* | | | | | | | | | |
| --- | --- | --- | --- | --- | --- | --- | --- | --- | --- |
|  | G | BEX | MD | CI | EI | MEX | CY | PE | I-ECV |
| BEX1 | **.70** | .30 | -.15 | -.08 | .04 | .05 | .11 | -.01 | **.78** |
| BEX2 | .03 | .48 | .18 | .03 | -.02 | .20 | .09 | -.24 | .00 |
| BEX3 | **.66** | .36 | -.04 | .09 | -.11 | .11 | -.05 | -.01 | **.72** |
| BEX4 | **.53** | **.49** | .05 | -.08 | .02 | .11 | -.12 | -.16 | .48 |
| BEX5 | **.72** | .14 | -.05 | .02 | -.15 | .17 | -.06 | .06 | **.86** |
| BEX6 | **.73** | .26 | .03 | .16 | -.04 | -.04 | .05 | .04 | **.84** |
| BEX7 | **.69** | .32 | .16 | .09 | -.04 | .02 | -.11 | -.04 | **.76** |
| BEX8 | **.70** | .26 | -.05 | -.06 | .02 | .17 | -.19 | -.16 | **.75** |
| MD1 | **.83** | -.07 | -.20 | -.03 | -.04 | -.16 | .07 | .01 | **.90** |
| MD2 | **.51** | .02 | -.03 | -.02 | .00 | -.17 | -.03 | .07 | **.87** |
| MD3 | **.49** | .00 | .31 | -.17 | .08 | -.16 | .10 | -.07 | **.58** |
| MD4 | **.63** | -.15 | .14 | -.16 | .07 | -.13 | -.07 | -.08 | **.80** |
| MD5 | .48 | .18 | .52 | .00 | .08 | -.05 | .14 | .04 | .41 |
| CI1 | **.67** | .03 | -.02 | **.47** | -.04 | -.02 | .09 | .08 | **.65** |
| CI2 | **.66** | .09 | .02 | **.53** | .04 | -.01 | .03 | .04 | **.60** |
| CI3 | **.63** | .03 | -.05 | **.51** | .07 | -.05 | -.04 | .04 | **.59** |
| CI4 | **.66** | .01 | -.01 | **.60** | .06 | .01 | .00 | .07 | **.54** |
| CI5 | **.46** | -.10 | -.09 | **.38** | .20 | .01 | -.12 | .18 | .45 |
| EI1 | **.47** | -.08 | -.13 | .11 | **.63** | -.11 | .05 | .09 | .32 |
| EI2 | **.41** | .00 | -.08 | .06 | **.56** | -.04 | .04 | .15 | .33 |
| EI3 | **.38** | -.13 | .10 | .07 | **.49** | .10 | -.16 | -.06 | .31 |
| EI4 | **.59** | .05 | .07 | -.08 | **.37** | .05 | .04 | .11 | **.68** |
| EI5 | .28 | .05 | .22 | .08 | **.60** | .02 | -.02 | .04 | .16 |
| MEX1 | **.72** | .10 | -.04 | -.04 | .04 | **.47** | .09 | -.08 | **.67** |
| MEX2 | **.72** | .12 | -.04 | -.01 | .07 | **.42** | -.07 | -.12 | **.71** |
| MEX3 | **.78** | .13 | .03 | .03 | -.09 | **.39** | -.02 | .04 | **.76** |
| MEX4 | **.73** | .06 | .00 | .03 | -.08 | **.37** | -.03 | -.15 | **.75** |
| MEX5 | **.72** | .20 | -.08 | -.07 | .02 | **.45** | .11 | -.10 | **.65** |
| CY1 | **.79** | -.14 | -.06 | -.03 | -.13 | .09 | .38 | .00 | **.76** |
| CY2 | **.80** | -.15 | -.19 | -.04 | -.12 | .06 | .38 | -.02 | **.75** |
| CY3 | .30 | -.05 | .14 | .08 | .01 | .06 | -.11 | -.23 | .48 |
| CY4 | .48 | .07 | .47 | .04 | .10 | -.05 | .33 | .01 | .39 |
| CY5 | **.54** | .00 | .16 | .07 | .20 | .05 | .20 | .34 | **.56** |
| PE1 | .14 | -.01 | -.08 | .03 | **.38** | -.02 | .09 | **.48** | .05 |
| PE2 | .33 | -.06 | .10 | -.05 | .14 | -.12 | .09 | **.54** | .24 |
| PE3 | .16 | .05 | -.07 | .19 | .14 | -.06 | -.06 | **.53** | .07 |
| PE4 | **.37** | -.25 | .01 | -.11 | -.06 | -.11 | .09 | **.42** | .33 |
| PE5 | **.40** | -.25 | .01 | -.02 | -.22 | -.05 | .05 | **.54** | .28 |
| PE6 | **.33** | .03 | .00 | **.22** | -.03 | .01 | -.06 | **.70** | .17 |
| *Note.* BEX = BAT Exhaustion, MD = Mental Distance, CI = Cognitive Impairment, EI = Emotional Impairment, MEX = MBI Exhaustion, CY = Cynicism, PE = Professional Efficacy, G = General Factor, I-ECV = item-explained common variance. Shown factor loadings are statistically significant at *p* < .001. Statistically significant factor loadings and item explained common variance > .50 in bold. Professional Efficacy items are reverse scored. | | | | | | | | | |

| **Supplementary Table OS25**  *Target Rotated Bifactor Model and Item Explained Common Variance for the BAT and MBI Items for the United States* | | | | | | | | | |
| --- | --- | --- | --- | --- | --- | --- | --- | --- | --- |
|  | G | BEX | MD | CI | EI | MEX | CY | PE | I-ECV |
| BEX1 | **.61** | **.39** | -.09 | .07 | -.04 | **.38** | -.03 | -.01 | **.54** |
| BEX2 | .08 | **.49** | .08 | -.03 | .11 | .22 | .04 | .00 | .02 |
| BEX3 | **.58** | **.43** | -.12 | .05 | .03 | **.27** | -.04 | -.01 | **.54** |
| BEX4 | **.52** | **.46** | .03 | -.08 | .06 | .07 | -.12 | -.06 | **.52** |
| BEX5 | **.70** | .14 | -.28 | -.03 | .01 | -.09 | -.03 | .07 | **.81** |
| BEX6 | **.64** | .21 | -.14 | .15 | .08 | -.28 | .13 | .08 | **.68** |
| BEX7 | **.59** | **.57** | .07 | .02 | .16 | -.21 | -.11 | .02 | .46 |
| BEX8 | **.62** | **.45** | -.19 | .02 | -.01 | .25 | -.03 | -.10 | **.55** |
| MD1 | **.84** | -.10 | .06 | .02 | **-.18** | -.10 | -.03 | .09 | **.91** |
| MD2 | **.56** | -.22 | .06 | .15 | -.07 | -.17 | -.24 | .03 | **.65** |
| MD3 | **.58** | -.06 | .39 | -.15 | .03 | .06 | .09 | .03 | **.63** |
| MD4 | **.60** | -.28 | .24 | .01 | -.15 | -.01 | .06 | .12 | **.66** |
| MD5 | **.54** | -.09 | .37 | .11 | -.01 | .00 | .31 | .00 | **.53** |
| CI1 | **.50** | -.14 | -.01 | **.70** | -.02 | .06 | -.02 | .04 | .33 |
| CI2 | **.51** | .03 | .09 | **.65** | .12 | .18 | .01 | .02 | .36 |
| CI3 | **.49** | .04 | .01 | **.67** | .03 | -.13 | -.02 | .10 | .33 |
| CI4 | **.51** | .03 | -.10 | **.72** | .02 | -.11 | .04 | -.01 | .33 |
| CI5 | **.34** | .14 | .04 | **.50** | **.26** | -.11 | -.06 | .06 | .25 |
| EI1 | **.39** | -.01 | .02 | .13 | **.71** | .03 | -.11 | -.04 | .22 |
| EI2 | **.39** | .01 | .05 | -.01 | **.64** | .04 | -.10 | .10 | .26 |
| EI3 | **.44** | .16 | .01 | .08 | **.51** | .04 | .12 | -.07 | .38 |
| EI4 | **.54** | .14 | -.14 | .08 | **.52** | .02 | .09 | .06 | .47 |
| EI5 | **.35** | .04 | -.03 | .08 | **.69** | -.06 | .03 | .02 | .20 |
| MEX1 | **.75** | .20 | -.01 | -.01 | .07 | **.46** | .00 | -.13 | **.68** |
| MEX2 | **.71** | **.28** | .03 | -.05 | -.01 | **.39** | -.06 | **-.20** | **.64** |
| MEX3 | **.81** | .16 | -.26 | -.10 | .04 | .15 | .10 | -.09 | **.82** |
| MEX4 | **.79** | **.25** | .03 | -.01 | .05 | .13 | .04 | -.09 | **.87** |
| MEX5 | **.76** | **.26** | .11 | -.08 | .00 | .29 | -.05 | -.09 | **.76** |
| CY1 | **.84** | -.16 | .22 | -.10 | -.09 | .03 | .07 | .04 | **.88** |
| CY2 | **.84** | -.15 | .23 | -.06 | -.07 | .01 | .09 | .05 | **.88** |
| CY3 | **.53** | -.02 | .10 | .11 | .00 | .04 | .05 | -.18 | **.82** |
| CY4 | **.72** | -.05 | .17 | .01 | .04 | -.03 | .45 | .04 | **.68** |
| CY5 | **.67** | -.14 | .06 | -.02 | -.04 | -.02 | **.50** | **.19** | **.59** |
| PE1 | -.11 | .12 | .13 | .12 | .07 | -.03 | .06 | **.52** | .04 |
| PE2 | **.32** | -.18 | .09 | .04 | -.10 | -.01 | .00 | **.65** | .17 |
| PE3 | .13 | .20 | .00 | .17 | .11 | .03 | .10 | **.72** | .03 |
| PE4 | **.40** | -.22 | .05 | -.20 | -.04 | -.10 | -.12 | **.46** | .33 |
| PE5 | **.39** | -.22 | -.03 | -.12 | -.06 | -.16 | .02 | **.61** | .25 |
| PE6 | .12 | .14 | -.10 | .11 | .07 | -.05 | .07 | **.77** | .02 |
| *Note.* BEX = BAT Exhaustion, MD = Mental Distance, CI = Cognitive Impairment, EI = Emotional Impairment, MEX = MBI Exhaustion, CY = Cynicism, PE = Professional Efficacy, G = General Factor, I-ECV = item-explained common variance. Shown factor loadings are statistically significant at *p* < .001. Statistically significant factor loadings and item explained common variance > .50 in bold. Professional Efficacy items are reverse scored. | | | | | | | | | |

**7. Bifactor indices for the BAT and MBI**

| **Supplementary Table OS26**  *Bifactor Indices for the Target Rotated Bifactor Models for the BAT and MBI* | | | | | | | | | |
| --- | --- | --- | --- | --- | --- | --- | --- | --- | --- |
| Sample | Index | G | BEX | MD | CI | EI | MEX | CY | PE |
| Combined | ECV | .57 | .07 | .03 | .08 | .07 | .05 | .04 | .10 |
|  | ω_h_ | .89 | .20 | .00 | .45 | .47 | .17 | .13 | .66 |
|  | ω_Rel._ | .92 | .20 | .00 | .47 | .49 | .18 | .13 | .68 |
|  | H | .96 | .54 | .21 | .73 | .70 | .46 | .39 | .77 |
| Australia | ECV | .58 | .06 | .03 | .08 | .06 | .05 | .06 | .09 |
|  | ω_h_ | .90 | .19 | .06 | .45 | .38 | .17 | .20 | .65 |
|  | ω_Rel._ | .92 | .20 | .06 | .46 | .39 | .18 | .21 | .66 |
|  | H | .97 | .55 | .29 | .74 | .67 | .48 | .58 | .77 |
| Netherlands | ECV | .57 | .06 | .04 | .07 | .07 | .05 | .05 | .08 |
|  | ω_h_ | .89 | .16 | .11 | .43 | .47 | .18 | .15 | .59 |
|  | ω_Rel._ | .92 | .16 | .11 | .44 | .48 | .19 | .16 | .61 |
|  | H | .96 | .48 | .37 | .68 | .70 | .46 | .51 | .74 |
| South Africa | ECV | .57 | .06 | .04 | .07 | .08 | .05 | .03 | .10 |
|  | ω_h_ | .89 | .20 | .04 | .35 | .50 | .23 | .11 | .60 |
|  | ω_Rel._ | .92 | .21 | .05 | .36 | .51 | .23 | .12 | .62 |
|  | H | .96 | .53 | .35 | .63 | .68 | .52 | .34 | .73 |
| United States | ECV | .51 | .09 | .03 | .10 | .09 | .05 | .03 | .11 |
|  | ω_h_ | .86 | .30 | .09 | .59 | .59 | .10 | .08 | .76 |
|  | ω_Rel._ | .88 | .31 | .09 | .61 | .60 | .11 | .08 | .78 |
|  | H | .96 | .64 | .29 | .80 | .77 | .37 | .37 | .82 |
| *Note.* G = General factor, BEX = BAT Exhaustion, MD = Mental Distance, CI = Cognitive Impairment, EI = Emotional Impairment, MEX = MBI Exhaustion, CY = Cynicism, PE = Professional Efficacy, ECV = explained common variance, ω_h_ = coefficient omega hierarchical, ω_Rel._ = relative omega, H = construct replicability. | | | | | | | | | |

**8. Category thresholds**

| **Supplementary Table OS27**  *Item Category Response Function for Australia, the Netherlands, South Africa, and the United States* | | | | | |
| --- | --- | --- | --- | --- | --- |
|  | Observed | Expected | Infit | Outfit | Andrich |
| Australia | | | | | |
| Never | -2.80 | -2.70 | .92 | .94 | . |
| Rarely | -1.34 | -1.40 | 1.01 | .96 | -2.69 |
| Sometimes | -.34 | -.35 | .99 | 1.11 | -.77 |
| Often | .52 | .59 | 1.10 | 1.11 | .93 |
| Always | 1.96 | 1.93 | .96 | .96 | 2.53 |
| Netherlands | | | | | |
| Never | -2.10 | -2.06 | .98 | .98 | . |
| Rarely | -1.13 | -1.18 | 1.04 | .99 | -2.14 |
| Sometimes | -.42 | -.40 | .97 | .97 | -.67 |
| Often | .31 | .35 | 1.04 | 1.05 | .83 |
| Always | 1.48 | 1.38 | .98 | 1.08 | 1.98 |
| South Africa | | | | | |
| Never | -2.04 | -1.99 | .98 | 1.01 | . |
| Rarely | -1.10 | -1.13 | .99 | .97 | -1.96 |
| Sometimes | -.36 | -.38 | .94 | .96 | -.60 |
| Often | .35 | .34 | .99 | .99 | .73 |
| Always | 1.13 | 1.27 | 1.17 | 1.19 | 1.83 |
| United States | | | | | |
| Never | -1.85 | -1.77 | .92 | .94 | . |
| Rarely | -.86 | -.92 | 1.10 | 1.06 | -2.05 |
| Sometimes | -.23 | -.22 | .97 | .97 | -.50 |
| Often | .39 | .41 | .99 | 1.00 | .77 |
| Always | 1.09 | 1.10 | 1.06 | 1.14 | 1.77 |
| *Note.* Observed = average observed logit, Expected = average model expected logit, Andrich = Rasch-Andrich thresholds. | | | | | |

**9. Item locations and fit statistics**

| **Supplementary Table O28**  *Rasch item locations and Fit Statistics for Australia* | | | | | | |
| --- | --- | --- | --- | --- | --- | --- |
| Item | Location | SE | Infit | Z_Infit_ | Outfit | Z_Outfit_ |
| EX1 | -1.08 | .10 | .68 [.56, .83] | -3.69 | .92 [.59, 1.54] | -.81 |
| EX2 | -1.21 | .10 | **1.24 [1.02, 1.49]** | 2.36 | **1.57 [1.12, 2.33]** | 4.99 |
| EX3 | -1.18 | .10 | .82 [.68, .98] | -1.91 | .81 [.68, .97] | -2.01 |
| EX4 | -.44 | .10 | 1.23 [1.00, 1.50] | 2.27 | **1.22 [.99, 1.48]** | 2.13 |
| EX5 | -.91 | .10 | .87 [.71, 1.05] | -1.41 | .87 [.70, 1.06] | -1.35 |
| EX6 | -.15 | .10 | .86 [.72, 1.02] | -1.50 | .85 [.71, 1.01] | -1.59 |
| EX7 | -.36 | .10 | .82 [.67, 1.00] | -1.89 | .82 [.68, 1.00] | -1.92 |
| EX8 | -1.53 | .10 | .94 [.76, 1.10] | -.60 | .92 [.74, 1.07] | -.75 |
| MD1 | -.66 | .10 | .94 [.77, 1.09] | -.61 | .93 [.77, 1.08] | -.72 |
| MD2 | -.43 | .10 | **1.43 [1.20, 1.66]** | 3.90 | **1.49 [1.25, 1.78]** | 4.35 |
| MD3 | .42 | .10 | 1.20 [.99, 1.44] | 1.94 | 1.18 [.98, 1.42] | 1.72 |
| MD4 | .05 | .10 | **1.30 [1.08, 1.56]** | 2.80 | **1.29 [1.06, 1.55]** | 2.72 |
| MD5 | .10 | .10 | **1.42 [1.22, 1.69]** | 3.81 | **1.39 [1.19, 1.64]** | 3.55 |
| CI1 | -.39 | .10 | .85 [.72, 1.01] | -1.59 | .83 [.70, .99] | -1.78 |
| CI2 | .29 | .10 | .71 [.59, .82] | -3.26 | .70 [.59, .80] | -3.30 |
| CI3 | .37 | .10 | .88 [.74, 1.05] | -1.22 | .89 [.73, 1.07] | -1.08 |
| CI4 | .10 | .10 | .83 [.69, .98] | -1.83 | .82 [.70, .98] | -1.86 |
| CI5 | .65 | .10 | .68 [.55, .80] | -3.63 | .71 [.58, .84] | -3.11 |
| EI1 | 1.53 | .11 | .91 [.69, 1.27] | -.89 | .92 [.71, 1.20] | -.64 |
| EI2 | 1.81 | .12 | 1.19 [.97, 1.41] | 1.74 | 1.04 [.85, 1.21] | .34 |
| EI3 | .33 | .10 | 1.14 [.96, 1.36] | 1.37 | 1.14 [.95, 1.39] | 1.35 |
| EI4 | 1.46 | .11 | 1.06 [.88, 1.26] | .64 | 1.05 [.84, 1.37] | .43 |
| EI5 | 1.23 | .11 | 1.08 [.90, 1.24] | .78 | 1.05 [.89, 1.24] | .52 |
| *Note.* EX = BAT Exhaustion, MD = Mental Distance, CI = Cognitive Impairment, EI = Emotional Impairment, SE = standard error of the location. 95% bias-corrected and accelerated confidence intervals in parentheses (1000 samples). Infit and outfit mean squares showing underfit in bold. | | | | | | |

| **Supplementary Table OS29**  *Rasch item locations and fit statistics for the Netherlands* | | | | | | |
| --- | --- | --- | --- | --- | --- | --- |
| Item | Location | SE | Infit | Z_Infit_ | Outfit | Z_Outfit_ |
| EX1 | -.69 | .09 | .65 [.53, .78] | -4.21 | .64 [.52, .77] | -4.24 |
| EX2 | -.90 | .09 | **1.37 [1.11, 1.65]** | 3.47 | **1.41 [1.15, 1.72]** | 3.81 |
| EX3 | -.96 | .09 | .87 [.73, 1.02] | -1.44 | .87 [.72, 1.01] | -1.36 |
| EX4 | -.24 | .09 | 1.14 [.96, 1.36] | 1.41 | 1.11 [.93, 1.32] | 1.15 |
| EX5 | -1.07 | .09 | .98 [.81, 1.2] | -.23 | .96 [.79, 1.17] | -.36 |
| EX6 | -.19 | .09 | .96 [.77, 1.13] | -.43 | .93 [.77, 1.10] | -.70 |
| EX7 | -.37 | .09 | .74 [.64, .87] | -2.90 | .73 [.61, .84] | -3.06 |
| EX8 | -1.10 | .09 | .87 [.74, 1.06] | -1.42 | .85 [.72, 1.03] | -1.56 |
| MD1 | -.47 | .09 | .91 [.77, 1.11] | -.91 | .90 [.75, 1.07] | -1.08 |
| MD2 | -.24 | .09 | **1.58 [1.38, 1.88]** | 5.13 | **1.61 [1.40, 1.91]** | 5.39 |
| MD3 | .61 | .10 | 1.12 [.88, 1.46] | 1.20 | 1.23 [.88, 1.85] | 2.23 |
| MD4 | .38 | .09 | 1.00 [.85, 1.19] | .01 | 1.03 [.87, 1.25] | .32 |
| MD5 | .28 | .09 | **1.48 [1.24, 1.73]** | 4.31 | **1.46 [1.25, 1.71]** | 4.20 |
| CI1 | -.41 | .09 | .87 [.73, 1.00] | -1.41 | .86 [.72, .98] | -1.46 |
| CI2 | .04 | .09 | .70 [.57, .83] | -3.45 | .68 [.57, .81] | -3.69 |
| CI3 | .14 | .09 | .70 [.56, .84] | -3.47 | .69 [.56, .81] | -3.60 |
| CI4 | -.12 | .09 | .72 [.60, .84] | -3.18 | .71 [.59, .82] | -3.35 |
| CI5 | .48 | .10 | .84 [.71, 1.00] | -1.65 | .87 [.72, 1.03] | -1.36 |
| EI1 | 1.32 | .11 | 1.04 [.81, 1.34] | .44 | .99 [.77, 1.26] | -.07 |
| EI2 | 1.39 | .11 | **1.32 [1.06, 1.61]** | 2.86 | 1.20 [.96, 1.49] | 1.73 |
| EI3 | -.05 | .09 | 1.11 [.93, 1.32] | 1.13 | 1.13 [.95, 1.35] | 1.34 |
| EI4 | 1.07 | .10 | 1.03 [.87, 1.19] | .33 | .94 [.80, 1.09] | -.59 |
| EI5 | 1.11 | .10 | **1.29 [1.08, 1.57]** | 2.65 | **1.28 [1.06, 1.54]** | 2.48 |
| *Note.* EX = BAT Exhaustion, MD = Mental Distance, CI = Cognitive Impairment, EI = Emotional Impairment, SE = standard error of the location. 95% bias-corrected and accelerated confidence intervals in parentheses (1000 samples). Infit and outfit mean squares showing underfit in bold. | | | | | | |

| **Supplementary Table OS30**  *Rasch item locations and fit statistics for South Africa* | | | | | | |
| --- | --- | --- | --- | --- | --- | --- |
| Item | Location | SE | Infit | Z_Infit_ | Outfit | Z_Outfit_ |
| EX1 | -.83 | .09 | .56 [.45, .69] | -5.58 | .57 [.45, .70] | -5.35 |
| EX2 | -1.89 | .09 | **2.04 [1.68, 2.43]** | 8.45 | **2.29 [1.82, 2.99]** | 9.47 |
| EX3 | -.50 | .09 | .79 [.67, .93] | -2.32 | .78 [.65, .90] | -2.50 |
| EX4 | -.58 | .09 | .79 [.63, 1.00] | -2.39 | .79 [.65, 1.02] | -2.32 |
| EX5 | -.67 | .09 | .89 [.74, 1.04] | -1.20 | .89 [.75, 1.04] | -1.12 |
| EX6 | -.13 | .09 | .70 [.56, .87] | -3.50 | .69 [.57, .87] | -3.50 |
| EX7 | -.36 | .09 | .83 [.68, 1.00] | -1.88 | .82 [.68, .98] | -2.01 |
| EX8 | -1.09 | .09 | .72 [.58, .86] | -3.31 | .72 [.59, .86] | -3.25 |
| MD1 | -.22 | .09 | .88 [.72, 1.03] | -1.32 | .86 [.73, 1.02] | -1.44 |
| MD2 | -.07 | .09 | **1.40 [1.19, 1.63]** | 3.70 | **1.39 [1.19, 1.65]** | 3.62 |
| MD3 | -.32 | .09 | **1.38 [1.13, 1.64]** | 3.60 | **1.39 [1.14, 1.64]** | 3.65 |
| MD4 | .16 | .09 | .97 [.79, 1.19] | -.27 | .96 [.78, 1.18] | -.35 |
| MD5 | -.23 | .09 | 1.24 [.98, 1.50] | 2.30 | **1.30 [1.01, 1.60]** | 2.87 |
| CI1 | .16 | .09 | .78 [.65, .94] | -2.37 | .78 [.64, .92] | -2.41 |
| CI2 | .42 | .09 | .69 [.58, .82] | -3.53 | .70 [.58, .84] | -3.32 |
| CI3 | .60 | .09 | .70 [.56, .84] | -3.30 | .70 [.57, .85] | -3.27 |
| CI4 | .45 | .09 | .72 [.63, .84] | -3.06 | .73 [.62, .83] | -2.90 |
| CI5 | .60 | .09 | .86 [.71, 1.03] | -1.44 | .92 [.74, 1.11] | -.81 |
| EI1 | 1.21 | .10 | 1.10 [.88, 1.32] | .96 | 1.00 [.83, 1.21] | .05 |
| EI2 | 1.20 | .10 | **1.44 [1.18, 1.68]** | 3.69 | **1.37 [1.11, 1.76]** | 2.93 |
| EI3 | -.07 | .09 | **1.39 [1.18, 1.64]** | 3.63 | **1.36 [1.14, 1.58]** | 3.31 |
| EI4 | 1.11 | .10 | 1.03 [.83, 1.23] | .34 | .92 [.77, 1.08] | -.74 |
| EI5 | 1.05 | .10 | **1.37 [1.15, 1.57]** | 3.21 | **1.33 [1.07, 1.61]** | 2.70 |
| *Note.* EX = BAT Exhaustion, MD = Mental Distance, CI = Cognitive Impairment, EI = Emotional Impairment, SE = standard error of the location. 95% bias-corrected and accelerated confidence intervals in parentheses (1000 samples). Infit and outfit mean squares showing underfit in bold. | | | | | | |

| **Supplementary Table OS31**  *Rasch item locations and fit statistics for the United States* | | | | | | |
| --- | --- | --- | --- | --- | --- | --- |
| Item | Location | SE | Infit | Z_Infit_ | Outfit | Z_Outfit_ |
| EX1 | -.86 | .08 | .69 [.55, .85] | -3.74 | .69 [.55, .83] | -3.67 |
| EX2 | -.94 | .08 | **1.50 [1.22, 1.82]** | 4.66 | **1.69 [1.30, 2.34]** | 6.11 |
| EX3 | -.74 | .08 | .90 [.74, 1.1] | -1.06 | .90 [.74, 1.10] | -1.07 |
| EX4 | -.38 | .09 | 1.12 [.93, 1.32] | 1.22 | **1.12 [.92, 1.33]** | 1.27 |
| EX5 | -.83 | .08 | .84 [.69, .99] | -1.72 | .86 [.68, 1.00] | -1.55 |
| EX6 | -.17 | .09 | .78 [.63, .95] | -2.48 | .77 [.64, .96] | -2.51 |
| EX7 | -.18 | .09 | .77 [.64, .90] | -2.6 | .76 [.65, .90] | -2.65 |
| EX8 | -1.01 | .08 | .78 [.64, .95] | -2.46 | .78 [.63, .92] | -2.49 |
| MD1 | -.57 | .08 | .89 [.74, 1.05] | -1.21 | .92 [.75, 1.10] | -.80 |
| MD2 | -.41 | .09 | **1.18 [1.02, 1.40]** | 1.81 | **1.20 [1.02, 1.44]** | 1.99 |
| MD3 | .43 | .09 | **1.42 [1.17, 1.73]** | 3.79 | **1.49 [1.18, 1.85]** | 4.31 |
| MD4 | .01 | .09 | **1.29 [1.07, 1.52]** | 2.81 | **1.33 [1.09, 1.58]** | 3.15 |
| MD5 | .13 | .09 | **1.42 [1.20, 1.66]** | 3.84 | **1.49 [1.23, 1.84]** | 4.42 |
| CI1 | -.26 | .09 | .99 [.83, 1.14] | -.12 | .96 [.82, 1.13] | -.39 |
| CI2 | .06 | .09 | .67 [.56, .79] | -3.81 | .66 [.54, .76] | -3.98 |
| CI3 | .25 | .09 | .82 [.68, .96] | -1.95 | .78 [.67, .93] | -2.37 |
| CI4 | .14 | .09 | .79 [.67, .93] | -2.31 | .77 [.64, .89] | -2.57 |
| CI5 | .55 | .09 | .79 [.65, .95] | -2.2 | .79 [.64, .94] | -2.3 |
| EI1 | 1.09 | .1 | 1.11 [.92, 1.31] | 1.11 | 1.06 [.88, 1.22] | .55 |
| EI2 | 1.45 | .1 | 1.19 [.98, 1.45] | 1.72 | 1.08 [.90, 1.28] | .73 |
| EI3 | .46 | .09 | 1.05 [.86, 1.26] | .49 | 1.02 [.84, 1.21] | .22 |
| EI4 | .69 | .09 | 1.04 [.88, 1.21] | .4 | .99 [.85, 1.14] | -.11 |
| EI5 | 1.1 | .1 | **1.22 [1.02, 1.45]** | 2.02 | 1.14 [.98, 1.36] | 1.32 |
| *Note.* EX = BAT Exhaustion, MD = Mental Distance, CI = Cognitive Impairment, EI = Emotional Impairment, SE = standard error of the location. 95% bias-corrected and accelerated confidence intervals in parentheses (1000 samples). Infit and outfit mean squares showing underfit in bold. | | | | | | |

**10. Item characteristic curves of misfitting items**

**Figure OS1**

*Item Characteristics Curve for EX2 in the Combined Sample*


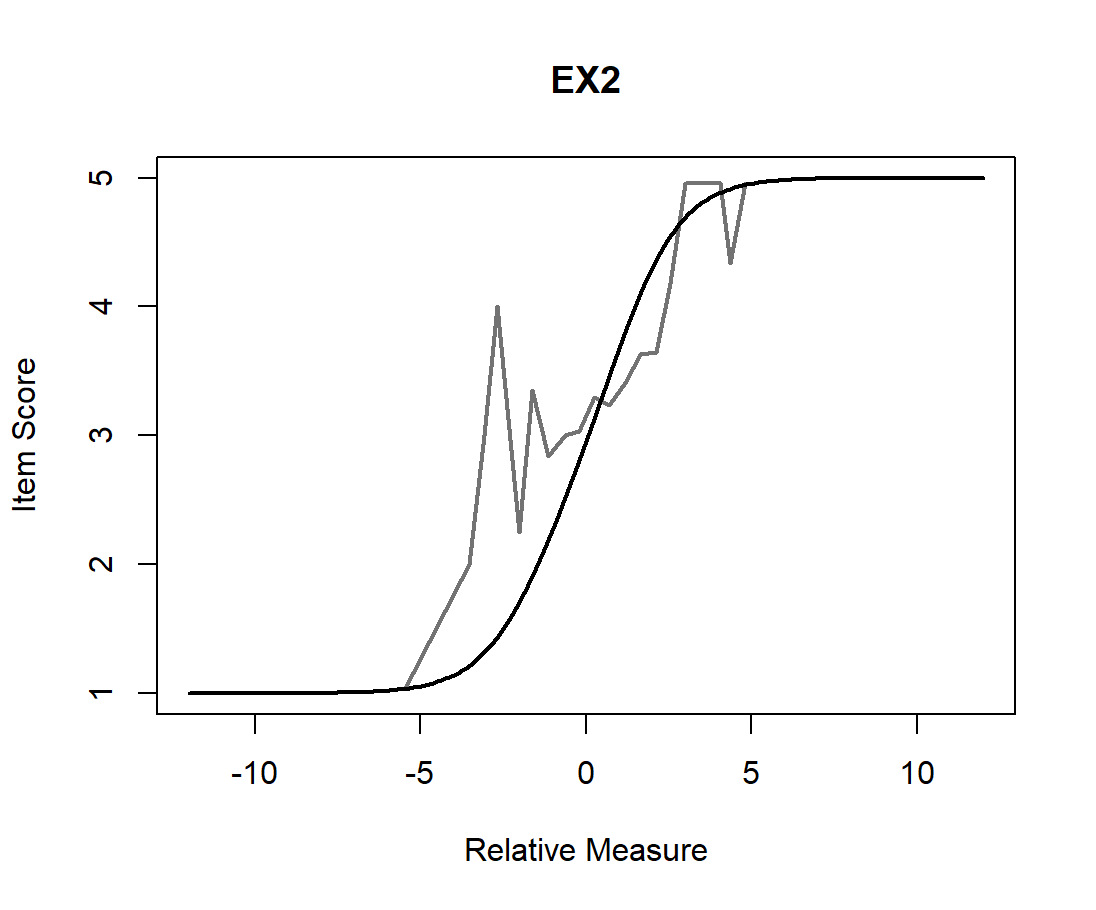


*Note*. Relative measure is the item characteristic curve relative to the item location. Black line is the Rasch expected item score. Grey line is the average observed item score.

**Figure OS2**

*Item Characteristics Curve for MD2 in the Combined Sample*


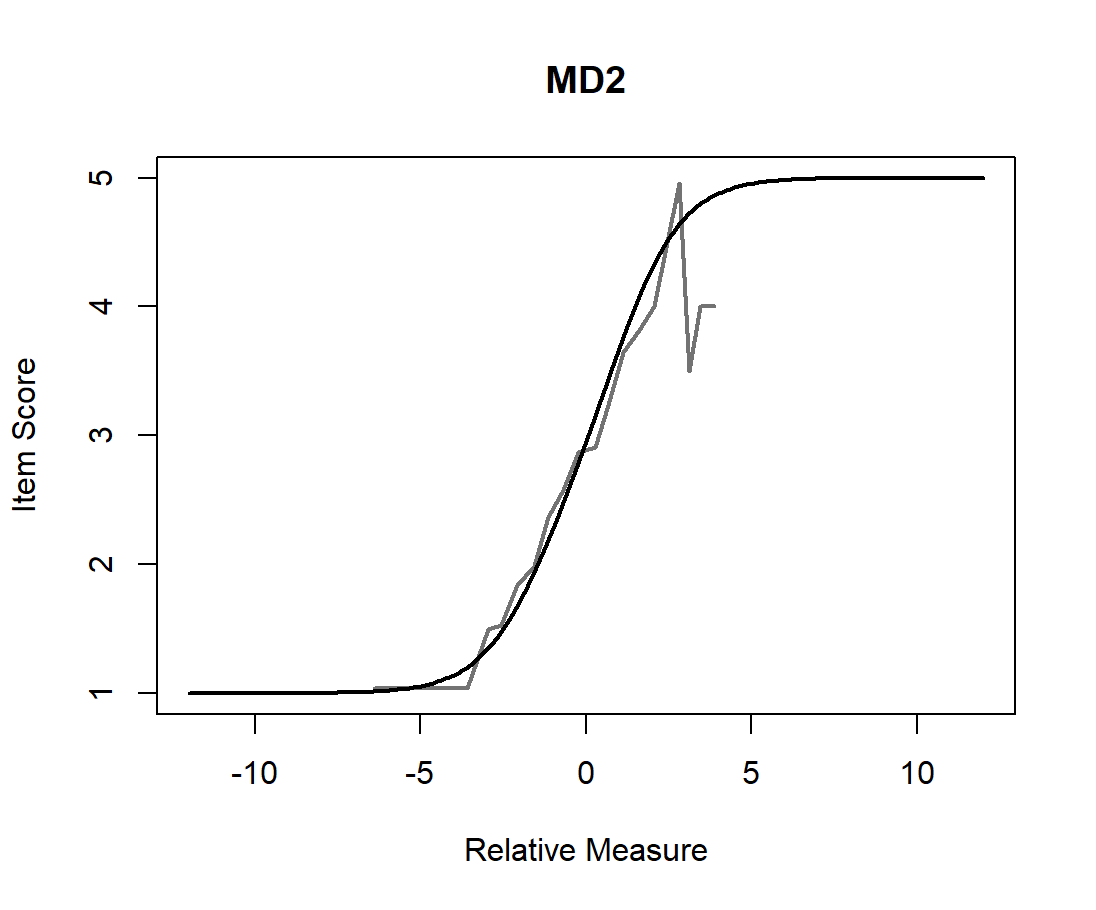


*Note*. Relative measure is the item characteristic curve relative to the item location. Black line is the Rasch expected item score. Grey line is the average observed item score.

**Figure OS3**

*Item Characteristics Curve for MD3 in the Combined Sample*


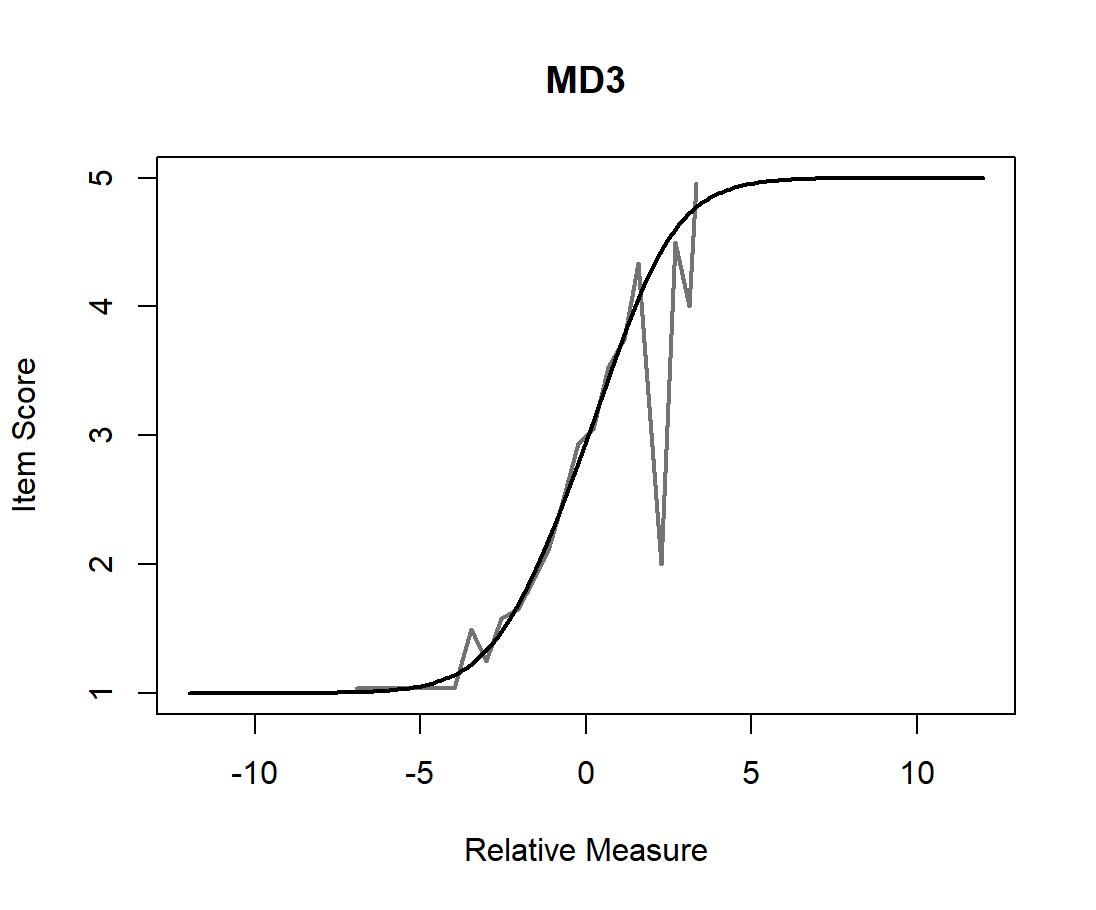


*Note*. Relative measure is the item characteristic curve relative to the item location. Black line is the Rasch expected item score. Grey line is the average observed item score.

**Figure OS4**

*Item Characteristics Curve for MD5 in the Combined Sample*


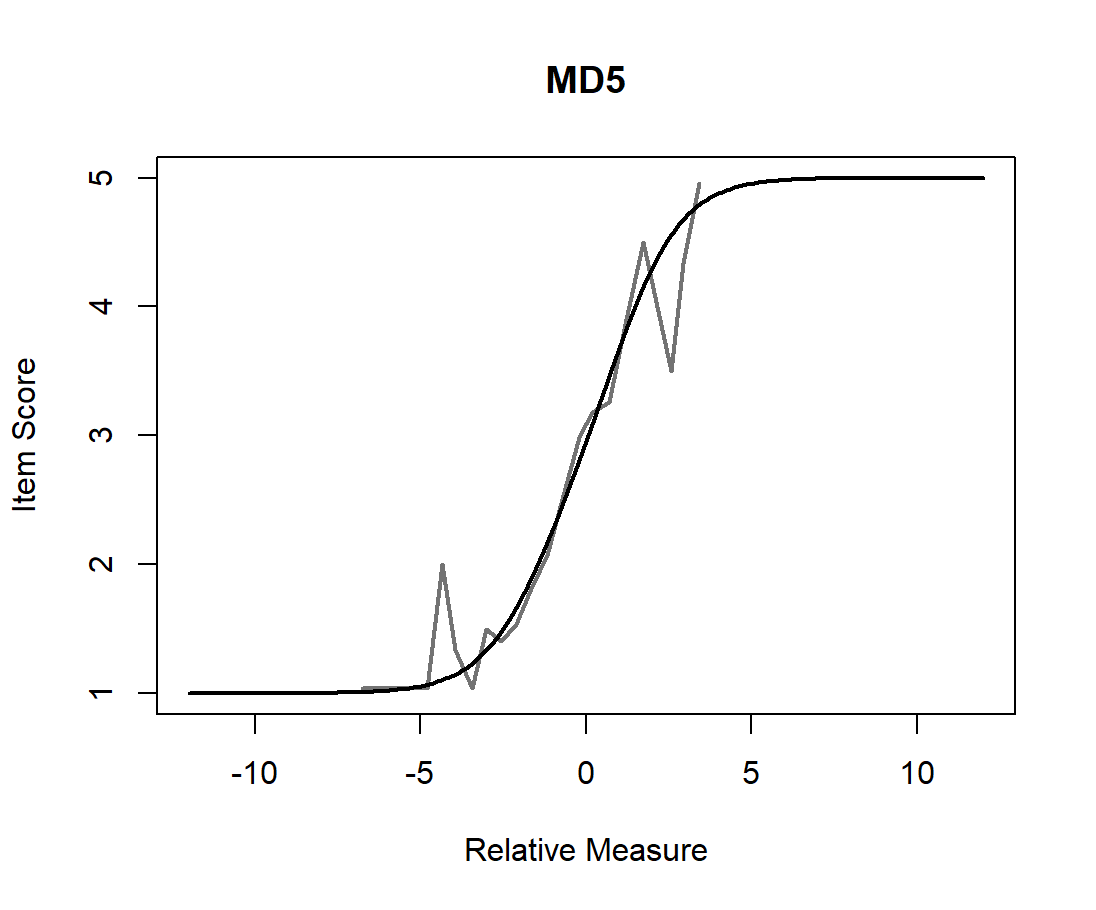


*Note*. Relative measure is the item characteristic curve relative to the item location. Black line is the Rasch expected item score. Grey line is the average observed item score.

**11. Wright maps**

**Figure OS5**

*Wright Map for the Combined Sample*

2 +

. |

|

. |

# |T EI2

. T| EI1

.# | EI5

1 ### + EI4

.## |

.### |S

.### | CI5

.###### |

.###### S| CI3 MD3

.######## | CI2 CI4 EI3 MD4

0 .######### +M MD5

.########### | CI1 EX6

.######### | EX7 MD2

########## | EX4 MD1

.############ |

.########### M|S

.############ | EX1 EX3 EX5

-1 .######### +

.########## | EX2 EX8

.######### |

.########## |T

.####### |

.### S|

.####### |

-2 .#### +

*Note*. Logits truncated between |2| logits. EX = Exhaustion, MD = Mental Distance, CI = Cognitive Impairment, EI = Emotional Impairment. Person measures presented on the left and item locations presented on the right.

**Figure OS6**

*Wright Map for Australia*

2 +

. |T EI2

. T|

. | EI1 EI4

. |

. | EI5

1 # +

# |S

### | CI5

.### | MD3

#### S| CI2 CI3 EI3

.### | CI4 MD5

0 ##### +M MD4

#### | EX6

###### | CI1 EX7

.####### | EX4 MD2

.#### | MD1

.##### M|S EX5

-1 ####### + EX1

##### | EX2 EX3

.##### |

# | EX8

.### |

.#### |T

-2 .### +

*Note*. Logits truncated between |2| logits. EX = Exhaustion, MD = Mental Distance, CI = Cognitive Impairment, EI = Emotional Impairment. Person measures presented on the left and item locations presented on the right.

**Figure OS7**

*Wright Map for the Netherlands*

2 +

. |

. |

|

. |T

| EI1 EI2

. T|

. | EI4 EI5

1 . +

|

# |S

# | MD3

## | CI5

## | MD4

.### S| MD5

.#### | CI3

0 #### +M CI2 EI3

#### | CI4 EX6

#### | EX4 MD2

#### | CI1 EX7

.###### | MD1

.#### | EX1

#### |S

####### M| EX2

-1 #### + EX3

.### | EX5 EX8

#### |

####### |

.# |T

### |

### |

#### S|

-2 ### +

*Note*. Logits truncated between |2| logits. EX = Exhaustion, MD = Mental Distance, CI = Cognitive Impairment, EI = Emotional Impairment. Person measures presented on the left and item locations presented on the right.

**Figure OS8**

*Wright Map for South Africa*

2 +

|

|

. |T

|

| EI1

# T| EI2 EI4

1 # + EI5

.# |

.# |S

.## | CI3 CI5

# | CI2 CI4

##### |

.### S| CI1 MD4

0 .## +M MD2

.##### | EI3 EX6

.##### | MD1 MD3 MD5

.##### | EX3 EX7

###### | EX4

######### |S EX5

###### M| EX1

-1 ### +

.##### | EX8

#### |

##### |

###### |T

.### |

.### S| EX2

-2 .# +

*Note*. Logits truncated between |2| logits. EX = Exhaustion, MD = Mental Distance, CI = Cognitive Impairment, EI = Emotional Impairment. Person measures presented on the left and item locations presented on the right.

**Figure OS9**

*Wright Map for the United States*

2 +

|

|

|

| EI2

# T|T

|

# | EI1 EI5

1 ## +

## |

.# |

.### |S EI4

## | CI5 EI3

## S| MD3

.## | CI3

######## | CI4 MD5

0 ######## +M CI2 MD4

.## | EX6 EX7

.##### | CI1

### | EX4 MD2

####### M|

.##### |S MD1

.##### | EX3

.## | EX1 EX5

-1 ##### + EX2 EX8

######## |

.#### |

# |T

#### S|

# |

.## |

|

-2 .## +

*Note*. Logits truncated between |2| logits. EX = Exhaustion, MD = Mental Distance, CI = Cognitive Impairment, EI = Emotional Impairment. Person measures presented on the left and item locations presented on the right.

**12. Differential item functioning**

| **Supplementary Table OS32**  *Chi-squared Difference Tests and Change in Nagelkerke R^2^ for Australia vs Other Countries* | | | | | | |
| --- | --- | --- | --- | --- | --- | --- |
| Item | *χ*^2^_M1M2_ | *χ*^2^_M1M3_ | *χ*^2^_M2M3_ | Δ*R*^2^_M1M2_ | Δ*R*^2^_M1M3_ | Δ*R*^2^_M2M3_ |
| EX1 | .365 | .080 | .039 | .001 | .004 | .003 |
| EX2 | .014 | .040 | .538 | .007 | .008 | .000 |
| EX3 | .013 | .045 | .864 | .004 | .004 | .000 |
| EX4 | .718 | .605 | .350 | .000 | .001 | .001 |
| EX5 | .121 | .240 | .504 | .002 | .002 | .000 |
| EX6 | .646 | .840 | .710 | .000 | .000 | .000 |
| EX7 | .908 | .991 | .946 | .000 | .000 | .000 |
| EX8 | .091 | .239 | .952 | .002 | .002 | .000 |
| MD1 | .132 | .307 | .767 | .001 | .001 | .000 |
| MD2 | .307 | .226 | .165 | .001 | .003 | .002 |
| MD3 | .490 | .128 | .057 | .000 | .004 | .003 |
| MD4 | .134 | .318 | .821 | .002 | .002 | .000 |
| MD5 | .948 | .897 | .645 | .000 | .000 | .000 |
| CI1 | .091 | .184 | .463 | .002 | .002 | .000 |
| CI2 | .477 | .775 | .948 | .000 | .000 | .000 |
| CI3 | .651 | .814 | .650 | .000 | .000 | .000 |
| CI4 | .386 | .645 | .723 | .000 | .001 | .000 |
| CI5 | .976 | .435 | .197 | .000 | .002 | .002 |
| EI1 | .927 | .977 | .845 | .000 | .000 | .000 |
| EI2 | .426 | .301 | .183 | .001 | .002 | .002 |
| EI3 | .121 | .183 | .320 | .002 | .003 | .001 |
| EI4 | .020 | .065 | .748 | .004 | .004 | .000 |
| EI5 | .341 | .229 | .153 | .001 | .003 | .002 |
| *Note*. *χ*^2^ = *p* values for the *χ*^2^ difference test, Δ*R*^2^ = *Change in Nagelkerke R^2^*. M1M2 = Model 1 vs Model 2 (uniform DIF), M1M3 = Model 1 vs Model 3 (total DIF), M2M3 = Model 2 vs Model 3 (non-uniform DIF). *p* values < .005 and change in *R*^2^ values > .035 are in bold. | | | | | | |

| **Supplementary Table OS33**  *Chi-squared Difference Tests and Change in Nagelkerke R^2^ for the Netherlands vs Other Countries* | | | | | | |
| --- | --- | --- | --- | --- | --- | --- |
| Item | *χ*^2^_M1M2_ | *χ*^2^_M1M3_ | *χ*^2^_M2M3_ | Δ*R*^2^_M1M2_ | Δ*R*^2^_M1M3_ | Δ*R*^2^_M2M3_ |
| EX1 | .005 | .018 | .778 | .006 | .006 | .000 |
| EX2 | **.000** | **.000** | .047 | .019 | .024 | .005 |
| EX3 | .035 | .107 | .819 | .003 | .003 | .000 |
| EX4 | .050 | .120 | .534 | .003 | .004 | .000 |
| EX5 | .002 | .004 | .188 | .006 | .008 | .001 |
| EX6 | .457 | .747 | .864 | .000 | .000 | .000 |
| EX7 | .242 | .467 | .696 | .001 | .001 | .000 |
| EX8 | .530 | .681 | .541 | .000 | .000 | .000 |
| MD1 | .830 | .875 | .638 | .000 | .000 | .000 |
| MD2 | .657 | .750 | .539 | .000 | .001 | .000 |
| MD3 | .**001** | **.003** | .674 | .010 | .010 | .000 |
| MD4 | .012 | .040 | .830 | .005 | .006 | .000 |
| MD5 | .035 | .050 | .212 | .004 | .005 | .001 |
| CI1 | **.001** | **.002** | .179 | .008 | .009 | .001 |
| CI2 | **.004** | .006 | .143 | .005 | .006 | .001 |
| CI3 | **.002** | .010 | .808 | .007 | .007 | .000 |
| CI4 | **.000** | **.000** | .347 | .013 | .013 | .001 |
| CI5 | .353 | .594 | .671 | .001 | .001 | .000 |
| EI1 | .387 | .648 | .727 | .001 | .001 | .000 |
| EI2 | .866 | .979 | .904 | .000 | .000 | .000 |
| EI3 | .013 | .025 | .277 | .006 | .007 | .001 |
| EI4 | .860 | .705 | .414 | .000 | .000 | .000 |
| EI5 | .674 | .591 | .350 | .000 | .001 | .001 |
| *Note*. *χ*^2^ = *p* values for the *χ*^2^ difference test, Δ*R*^2^ = *Change in Nagelkerke R^2^*. M1M2 = Model 1 vs Model 2 (uniform DIF), M1M3 = Model 1 vs Model 3 (total DIF), M2M3 = Model 2 vs Model 3 (non-uniform DIF). *p* values < .005 and change in *R*^2^ values > .035 are in bold. | | | | | | |

| **Supplementary Table OS34**  *Chi-squared Difference Tests and Change in Nagelkerke R^2^ for South Africa vs Other Countries* | | | | | | |
| --- | --- | --- | --- | --- | --- | --- |
| Item | *χ*^2^_M1M2_ | *χ*^2^_M1M3_ | *χ*^2^_M2M3_ | Δ*R*^2^_M1M2_ | Δ*R*^2^_M1M3_ | Δ*R*^2^_M2M3_ |
| EX1 | .775 | .734 | .463 | .000 | .000 | .000 |
| EX2 | **.000** | **.000** | .157 | **.080** | **.082** | .002 |
| EX3 | **.000** | **.000** | .926 | .011 | .011 | .000 |
| EX4 | .024 | .059 | .449 | .004 | .005 | .000 |
| EX5 | .120 | .298 | .992 | .002 | .002 | .000 |
| EX6 | .906 | .668 | .373 | .000 | .000 | .000 |
| EX7 | .267 | .210 | .169 | .001 | .002 | .001 |
| EX8 | .891 | .914 | .689 | .000 | .000 | .000 |
| MD1 | **.004** | .016 | .890 | .005 | .005 | .000 |
| MD2 | **.004** | **.004** | .091 | .008 | .011 | .003 |
| MD3 | **.000** | **.000** | .116 | **.037** | **.039** | .002 |
| MD4 | .884 | .963 | .816 | .000 | .000 | .000 |
| MD5 | **.002** | .005 | .234 | .008 | .009 | .001 |
| CI1 | **.000** | **.000** | .067 | .021 | .023 | .002 |
| CI2 | **.000** | **.000** | .136 | .010 | .012 | .001 |
| CI3 | **.000** | **.000** | .410 | .013 | .013 | .000 |
| CI4 | **.000** | **.000** | .072 | .016 | .018 | .002 |
| CI5 | .274 | .204 | .159 | .001 | .003 | .002 |
| EI1 | .607 | .867 | .883 | .000 | .000 | .000 |
| EI2 | .272 | .534 | .829 | .001 | .001 | .000 |
| EI3 | .012 | .033 | .495 | .006 | .006 | .000 |
| EI4 | .261 | .530 | .941 | .001 | .001 | .000 |
| EI5 | .871 | .703 | .410 | .000 | .001 | .001 |
| *Note.* χ^2^ = *p* values for the χ^2^ difference test, Δ*R*^2^ = Change in Nagelkerke *R*^2^. M1M2 = Model 1 vs Model 2 (uniform DIF), M1M3 = Model 1 vs Model 3 (total DIF), M2M3 = Model 2 vs Model 3 (non-uniform DIF). *p* values < .005 and change in *R*^2^ values > .035 are in bold. | | | | | | |

| **Supplementary Table OS35**  *Chi-squared Difference Tests and Change in Nagelkerke R^2^ for the United States vs Other Countries* | | | | | | |
| --- | --- | --- | --- | --- | --- | --- |
| Item | *χ*^2^_M1M2_ | *χ*^2^_M1M3_ | *χ*^2^_M2M3_ | Δ*R*^2^_M1M2_ | Δ*R*^2^_M1M3_ | Δ*R*^2^_M2M3_ |
| EX1 | .105 | .191 | .411 | .002 | .002 | .000 |
| EX2 | .195 | .350 | .517 | .002 | .003 | .000 |
| EX3 | .485 | .746 | .752 | .000 | .000 | .000 |
| EX4 | .987 | .543 | .269 | .000 | .001 | .001 |
| EX5 | .949 | .846 | .565 | .000 | .000 | .000 |
| EX6 | .876 | .573 | .296 | .000 | .001 | .001 |
| EX7 | .030 | .081 | .590 | .003 | .003 | .000 |
| EX8 | .226 | .472 | .845 | .001 | .001 | .000 |
| MD1 | .272 | .480 | .610 | .001 | .001 | .000 |
| MD2 | .030 | .094 | .954 | .005 | .005 | .000 |
| MD3 | .019 | .058 | .682 | .005 | .005 | .000 |
| MD4 | .228 | .438 | .656 | .001 | .001 | .000 |
| MD5 | .283 | .467 | .543 | .001 | .001 | .000 |
| CI1 | .777 | .872 | .660 | .000 | .000 | .000 |
| CI2 | .070 | .192 | .920 | .002 | .002 | .000 |
| CI3 | .519 | .794 | .831 | .000 | .000 | .000 |
| CI4 | .639 | .885 | .879 | .000 | .000 | .000 |
| CI5 | .907 | .922 | .701 | .000 | .000 | .000 |
| EI1 | .203 | .429 | .791 | .002 | .002 | .000 |
| EI2 | .668 | .620 | .380 | .000 | .001 | .001 |
| EI3 | **.001** | **.001** | .119 | .010 | .013 | .002 |
| EI4 | **.001** | **.004** | .374 | .008 | .008 | .001 |
| EI5 | .701 | .900 | .801 | .000 | .000 | .000 |
| *Note.* χ^2^ = *p* values for the χ^2^ difference test, Δ*R*^2^ = Change in Nagelkerke *R*^2^. M1M2 = Model 1 vs Model 2 (uniform DIF), M1M3 = Model 1 vs Model 3 (total DIF), M2M3 = Model 2 vs Model 3 (non-uniform DIF). *p* values < .005 and change in *R*^2^ values > .035 are in bold. | | | | | | |

| **Supplementary Table OS36**  *Chi-squared Difference Tests and Change in Nagelkerke R^2^ for Australia vs the Netherlands* | | | | | | |
| --- | --- | --- | --- | --- | --- | --- |
| Item | *χ*^2^_M1M2_ | *χ*^2^_M1M3_ | *χ*^2^_M2M3_ | Δ*R*^2^_M1M2_ | Δ*R*^2^_M1M3_ | Δ*R*^2^_M2M3_ |
| EX1 | .019 | .025 | .170 | .008 | .011 | .003 |
| EX2 | .295 | .285 | .235 | .002 | .006 | .003 |
| EX3 | .843 | .981 | .994 | .000 | .000 | .000 |
| EX4 | .330 | .409 | .360 | .002 | .003 | .002 |
| EX5 | **.003** | .005 | .199 | .011 | .013 | .002 |
| EX6 | .455 | .722 | .762 | .001 | .001 | .000 |
| EX7 | .412 | .704 | .866 | .001 | .001 | .000 |
| EX8 | .168 | .372 | .778 | .002 | .003 | .000 |
| MD1 | .410 | .706 | .896 | .001 | .001 | .000 |
| MD2 | .344 | .605 | .738 | .002 | .002 | .000 |
| MD3 | .079 | .124 | .296 | .004 | .006 | .002 |
| MD4 | .012 | .043 | .851 | .011 | .011 | .000 |
| MD5 | .168 | .225 | .299 | .003 | .005 | .002 |
| CI1 | .308 | .560 | .727 | .002 | .002 | .000 |
| CI2 | .028 | .065 | .427 | .006 | .007 | .001 |
| CI3 | .116 | .287 | .872 | .004 | .004 | .000 |
| CI4 | .033 | .099 | .768 | .006 | .006 | .000 |
| CI5 | .548 | .468 | .282 | .001 | .002 | .002 |
| EI1 | .526 | .811 | .901 | .001 | .001 | .000 |
| EI2 | .522 | .518 | .341 | .001 | .002 | .002 |
| EI3 | .009 | .016 | .226 | .012 | .014 | .002 |
| EI4 | .137 | .315 | .751 | .003 | .004 | .000 |
| EI5 | .381 | .210 | .125 | .002 | .006 | .004 |
| *Note.* χ^2^ = *p* values for the χ^2^ difference test, Δ*R*^2^ = Change in Nagelkerke *R*^2^. M1M2 = Model 1 vs Model 2 (uniform DIF), M1M3 = Model 1 vs Model 3 (total DIF), M2M3 = Model 2 vs Model 3 (non-uniform DIF). *p* values < .005 and change in *R*^2^ values > .035 are in bold. | | | | | | |

| **Supplementary Table OS37**  *Chi-squared Difference Tests and Change in Nagelkerke R^2^ for Australia vs South Africa* | | | | | | |
| --- | --- | --- | --- | --- | --- | --- |
| Item | *χ*^2^_M1M2_ | *χ*^2^_M1M3_ | *χ*^2^_M2M3_ | Δ*R*^2^_M1M2_ | Δ*R*^2^_M1M3_ | Δ*R*^2^_M2M3_ |
| EX1 | .676 | .183 | .073 | .000 | .005 | .004 |
| EX2 | **.000** | **.000** | .228 | **.108** | **.111** | .003 |
| EX3 | **.000** | **.000** | .909 | .022 | .022 | .000 |
| EX4 | .103 | .259 | .840 | .004 | .005 | .000 |
| EX5 | .960 | .929 | .704 | .000 | .000 | .000 |
| EX6 | .836 | .923 | .732 | .000 | .000 | .000 |
| EX7 | .416 | .468 | .354 | .001 | .002 | .001 |
| EX8 | .329 | .596 | .773 | .001 | .001 | .000 |
| MD1 | .007 | .025 | .829 | .008 | .008 | .000 |
| MD2 | .016 | .010 | .066 | .011 | .018 | .006 |
| MD3 | .000 | **.000** | .040 | **.034** | **.040** | .007 |
| MD4 | .301 | .567 | .800 | .002 | .002 | .000 |
| MD5 | .057 | .151 | .688 | .006 | .006 | .000 |
| CI1 | **.000** | **.000** | .114 | .027 | .030 | .003 |
| CI2 | .040 | .078 | .344 | .005 | .006 | .001 |
| CI3 | **.004** | .012 | .474 | .012 | .013 | .001 |
| CI4 | **.000** | **.001** | .178 | .016 | .018 | .002 |
| CI5 | .462 | .169 | .083 | .001 | .006 | .005 |
| EI1 | .700 | .911 | .845 | .000 | .000 | .000 |
| EI2 | .222 | .324 | .382 | .003 | .004 | .002 |
| EI3 | .012 | .027 | .332 | .011 | .013 | .002 |
| EI4 | .484 | .766 | .835 | .001 | .001 | .000 |
| EI5 | .536 | .318 | .167 | .001 | .004 | .004 |
| *Note.* χ^2^ = *p* values for the χ^2^ difference test, Δ*R*^2^ = Change in Nagelkerke R^2^. M1M2 = Model 1 vs Model 2 (uniform DIF), M1M3 = Model 1 vs Model 3 (total DIF), M2M3 = Model 2 vs Model 3 (non-uniform DIF). *p* values < .005 and change in *R*^2^ values > .035 are in bold. | | | | | | |

| **Supplementary Table OS38**  *Chi-squared Difference Tests and Change in Nagelkerke R^2^ for Australia vs the United States* | | | | | | |
| --- | --- | --- | --- | --- | --- | --- |
| Item | *χ*^2^_M1M2_ | *χ*^2^_M1M3_ | *χ*^2^_M2M3_ | Δ*R*^2^_M1M2_ | Δ*R*^2^_M1M3_ | Δ*R*^2^_M2M3_ |
| EX1 | .605 | .214 | .093 | .000 | .005 | .004 |
| EX2 | .502 | .567 | .408 | .001 | .003 | .002 |
| EX3 | .048 | .134 | .759 | .005 | .005 | .000 |
| EX4 | .823 | .459 | .219 | .000 | .003 | .003 |
| EX5 | .266 | .536 | .914 | .002 | .002 | .000 |
| EX6 | .809 | .687 | .405 | .000 | .001 | .001 |
| EX7 | .199 | .426 | .815 | .002 | .002 | .000 |
| EX8 | .078 | .210 | .908 | .004 | .004 | .000 |
| MD1 | .790 | .866 | .641 | .000 | .000 | .000 |
| MD2 | .409 | .536 | .452 | .001 | .002 | .001 |
| MD3 | .244 | .215 | .190 | .002 | .005 | .003 |
| MD4 | .915 | .973 | .837 | .000 | .000 | .000 |
| MD5 | .474 | .595 | .470 | .001 | .002 | .001 |
| CI1 | .396 | .573 | .530 | .001 | .002 | .001 |
| CI2 | .113 | .284 | .931 | .003 | .003 | .000 |
| CI3 | .871 | .986 | .961 | .000 | .000 | .000 |
| CI4 | .434 | .733 | .924 | .001 | .001 | .000 |
| CI5 | .989 | .902 | .649 | .000 | .000 | .000 |
| EI1 | .461 | .734 | .783 | .001 | .001 | .000 |
| EI2 | .812 | .416 | .193 | .000 | .003 | .003 |
| EI3 | .196 | .360 | .539 | .003 | .003 | .001 |
| EI4 | **.001** | **.002** | .466 | .016 | .017 | .001 |
| EI5 | .365 | .539 | .518 | .002 | .002 | .001 |
| *Note.* χ^2^ = *p* values for the χ^2^ difference test, ΔR^2^ = Change in Nagelkerke *R*^2^. M1M2 = Model 1 vs Model 2 (uniform DIF), M1M3 = Model 1 vs Model 3 (total DIF), M2M3 = Model 2 vs Model 3 (non-uniform DIF). *p* values < .005 and change in *R*^2^ values > .035 are in bold. | | | | | | |

| **Supplementary Table OS39**  *Chi-squared Difference Tests and Change in Nagelkerke R^2^ for the Netherlands vs South Africa* | | | | | | |
| --- | --- | --- | --- | --- | --- | --- |
| Item | *χ*^2^_M1M2_ | *χ*^2^_M1M3_ | *χ*^2^_M2M3_ | Δ*R*^2^_M1M2_ | Δ*R*^2^_M1M3_ | Δ*R*^2^_M2M3_ |
| EX1 | .052 | .142 | .730 | .005 | .005 | .000 |
| EX2 | **.000** | **.000** | .086 | **.116** | **.122** | .006 |
| EX3 | **.000** | **.001** | .962 | .019 | .019 | .000 |
| EX4 | .007 | .021 | .460 | .012 | .013 | .001 |
| EX5 | **.006** | .015 | .379 | .010 | .012 | .001 |
| EX6 | .581 | .712 | .540 | .000 | .001 | .000 |
| EX7 | .962 | .563 | .284 | .000 | .001 | .001 |
| EX8 | .643 | .743 | .538 | .000 | .001 | .000 |
| MD1 | .061 | .161 | .715 | .004 | .004 | .000 |
| MD2 | .158 | .154 | .185 | .004 | .008 | .004 |
| MD3 | **.000** | **.000** | .369 | **.065** | **.066** | .001 |
| MD4 | .135 | .326 | .980 | .004 | .004 | .000 |
| MD5 | **.002** | **.003** | .149 | .017 | .021 | .004 |
| CI1 | **.000** | **.000** | .093 | **.040** | **.044** | .004 |
| CI2 | **.000** | **.000** | .109 | .022 | .025 | .003 |
| CI3 | **.000** | **.000** | .649 | .028 | .028 | .000 |
| CI4 | **.000** | **.000** | .169 | **.042** | **.044** | .002 |
| CI5 | .220 | .347 | .435 | .003 | .004 | .001 |
| EI1 | .845 | .933 | .751 | .000 | .000 | .000 |
| EI2 | .585 | .858 | .925 | .001 | .001 | .000 |
| EI3 | .943 | .976 | .834 | .000 | .000 | .000 |
| EI4 | .440 | .634 | .574 | .001 | .001 | .000 |
| EI5 | .926 | .996 | .995 | .000 | .000 | .000 |
| *Note.* χ^2^ = *p* values for the χ^2^ difference test, Δ*R*^2^ = Change in Nagelkerke R^2^. M1M2 = Model 1 vs Model 2 (uniform DIF), M1M3 = Model 1 vs Model 3 (total DIF), M2M3 = Model 2 vs Model 3 (non-uniform DIF). *p* values < .005 and change in *R*^2^ values > .035 are in bold. | | | | | | |

| **Supplementary Table OS40**  *Chi-squared Difference Tests and Change in Nagelkerke R^2^ for the Netherlands vs the United States* | | | | | | |
| --- | --- | --- | --- | --- | --- | --- |
| Item | *χ*^2^_M1M2_ | *χ*^2^_M1M3_ | *χ*^2^_M2M3_ | Δ*R*^2^_M1M2_ | Δ*R*^2^_M1M3_ | Δ*R*^2^_M2M3_ |
| EX1 | .011 | .038 | .745 | .009 | .010 | .000 |
| EX2 | .122 | .090 | .119 | .006 | .012 | .006 |
| EX3 | .097 | .238 | .729 | .004 | .004 | .000 |
| EX4 | .335 | .604 | .780 | .002 | .002 | .000 |
| EX5 | .056 | .087 | .267 | .005 | .007 | .002 |
| EX6 | .656 | .795 | .610 | .000 | .001 | .000 |
| EX7 | .053 | .154 | .948 | .005 | .005 | .000 |
| EX8 | .689 | .844 | .673 | .000 | .000 | .000 |
| MD1 | .602 | .755 | .590 | .000 | .001 | .000 |
| MD2 | .107 | .258 | .736 | .005 | .006 | .000 |
| MD3 | .557 | .785 | .710 | .001 | .001 | .000 |
| MD4 | .026 | .078 | .667 | .009 | .009 | .000 |
| MD5 | .670 | .878 | .779 | .000 | .000 | .000 |
| CI1 | .090 | .147 | .330 | .005 | .006 | .002 |
| CI2 | .613 | .655 | .442 | .000 | .001 | .001 |
| CI3 | .155 | .363 | .969 | .003 | .003 | .000 |
| CI4 | .005 | .019 | .700 | .012 | .012 | .000 |
| CI5 | .627 | .792 | .632 | .000 | .001 | .000 |
| EI1 | .200 | .420 | .761 | .003 | .003 | .000 |
| EI2 | .739 | .823 | .598 | .000 | .001 | .001 |
| EI3 | **.000** | **.000** | .084 | .024 | .030 | .005 |
| EI4 | .076 | .139 | .374 | .005 | .006 | .001 |
| EI5 | .951 | .776 | .478 | .000 | .001 | .001 |
| *Note.* χ^2^ = *p* values for the χ^2^ difference test, Δ*R*^2^ = Change in Nagelkerke R^2^. M1M2 = Model 1 vs Model 2 (uniform DIF), M1M3 = Model 1 vs Model 3 (total DIF), M2M3 = Model 2 vs Model 3 (non-uniform DIF). *p* values < .005 and change in *R*^2^ values > .035 are in bold. | | | | | | |

| **Supplementary Table OS41**  *Chi-squared Difference Tests and Change in Nagelkerke R^2^ for South African vs the United States* | | | | | | |
| --- | --- | --- | --- | --- | --- | --- |
| Item | *χ*^2^_M1M2_ | *χ*^2^_M1M3_ | *χ*^2^_M2M3_ | Δ*R*^2^_M1M2_ | Δ*R*^2^_M1M3_ | Δ*R*^2^_M2M3_ |
| EX1 | .475 | .773 | .949 | .001 | .001 | .000 |
| EX2 | **.000** | **.000** | .743 | **.072** | **.072** | .000 |
| EX3 | .057 | .160 | .849 | .005 | .005 | .000 |
| EX4 | .170 | .209 | .264 | .003 | .005 | .002 |
| EX5 | .311 | .584 | .829 | .002 | .002 | .000 |
| EX6 | .996 | .439 | .199 | .000 | .002 | .002 |
| EX7 | .042 | .076 | .313 | .005 | .007 | .001 |
| EX8 | .430 | .724 | .881 | .001 | .001 | .000 |
| MD1 | .020 | .065 | .767 | .007 | .007 | .000 |
| MD2 | **.003** | .008 | .301 | .016 | .019 | .002 |
| MD3 | **.000** | **.000** | .584 | **.049** | **.050** | .001 |
| MD4 | .406 | .627 | .622 | .001 | .002 | .000 |
| MD5 | .015 | .027 | .262 | .011 | .013 | .002 |
| CI1 | **.001** | **.003** | .333 | .015 | .016 | .001 |
| CI2 | **.001** | **.002** | .346 | .015 | .016 | .001 |
| CI3 | **.003** | .010 | .423 | .013 | .014 | .001 |
| CI4 | .012 | .021 | .220 | .009 | .011 | .002 |
| CI5 | .414 | .378 | .258 | .001 | .004 | .002 |
| EI1 | .315 | .603 | .971 | .002 | .002 | .000 |
| EI2 | .405 | .647 | .675 | .001 | .002 | .000 |
| EI3 | **.000** | **.001** | .160 | .024 | .027 | .004 |
| EI4 | .012 | .038 | .680 | .009 | .010 | .000 |
| EI5 | .944 | .807 | .515 | .000 | .001 | .001 |
| *Note.* χ^2^ = *p* values for the χ^2^ difference test, Δ*R*^2^ = Change in Nagelkerke R^2^. M1M2 = Model 1 vs Model 2 (uniform DIF), M1M3 = Model 1 vs Model 3 (total DIF), M2M3 = Model 2 vs Model 3 (non-uniform DIF). *p* values < .005 and change in *R*^2^ values > .035 are in bold. | | | | | | |
